# Trends in stomatal density and size in maize hybrids representing 100 years of long-term breeding for yield

**DOI:** 10.3389/fpls.2026.1829321

**Published:** 2026-05-13

**Authors:** Memiş Bilgici, Elnaz Ebrahimi, Leticia Prada de Miranda, Sara Lira, Lucas Borras, Thomas Young, Recep Yavuz, Kenneth J. Moore, Philip Dixon, Thomas Lübberstedt

**Affiliations:** 1 Department of Agronomy, Iowa State University, Ames, IA, United States; 2 Department of Agronomy, Universidade Federal de Viçosa, Viçosa, MG, Brazil; 3 Corteva Agriscience, Johnston, IA, United States; 4 Department of Statistics, Iowa State University, Ames, IA, United States

**Keywords:** atmospheric CO_2_ concentration, average temperature anomalies (°C), climate change, hybrids, long-term breeding, maize, pioneer ERA hybrids, stomata

## Abstract

Maize hybrid breeding started over 100 years ago and has increased yield and vigor through improved genetics in conjunction with increased fertilizer and pesticide use, higher planting density, and agricultural mechanization. Stomata are expected to change in response to rising atmospheric CO_2_ concentration and average temperature anomalies (°C). Yet, the impact of long-term maize breeding over the past century on stomatal traits and their responses to climate factors remains poorly understood. We evaluated stomatal traits at the seedling stage in 27 Pioneer maize hybrids released from 1920 to 2022, grown under controlled conditions. Modern hybrids (2013-2022) had a smaller total stomatal pore area (9.17 × 10^8^ µm² leaf^−^¹ vs. 9.94 × 10^8^ µm² leaf^−^¹; *p* < 0.05), higher stomatal density (47.2 vs. 44.5 stomata mm^−^²), and smaller leaf area (17.9 vs. 20.5 cm²) than historical hybrids (1920-2011). Stomatal size (μm²), length (μm), and width (μm) did not differ significantly between the two groups. Across all hybrids, stomatal density was negatively correlated with stomatal size, length, width, and leaf area (r = -0.54 to -0.62). Total stomatal pore area declined in the hybrid’s year of release (r = -0.56, *p* < 0.05), whereas stomatal density increased over time (r = 0.51, *p* < 0.05). Stomatal traits were associated with release year and with release-year climate proxy variables (CO_2_ and °C) under a shared contemporary growth environment. We found that stomatal density was positively correlated with atmospheric CO_2_ concentration and temperature anomaly °C, whereas total stomatal pore area was negatively correlated with both variables. Our results indicate that modern maize hybrids differ from historical hybrids in stomatal density (increased), and total stomatal pore area (decreased), consistent with long-term breeding progress of inadvertent selection under changing production environments.

## Highlights

- Modern hybrids had higher stomatal density but lower total stomatal pore area than historical hybrids.
- Across 27 Pioneer hybrids, stomatal density was negatively correlated with stomatal size, length, width and leaf area.
- Over the past 100 years, the total stomatal pore area on leaves decreased, while stomatal density increased as leaf area declined, revealing a connection between these two patterns.
- Total stomatal pore area was negatively correlated with release-year atmospheric CO_2_ concentration and temperature anomaly (°C) over the past century.

## 1 Introduction

Maize hybrid breeding started over 100 years ago (Shull, 1908; East, 1908) and resulted in significant yield increases. Double-cross hybrids (crosses between two single-cross hybrids) made maize hybrids economically viable (Jones, 1922). In 1926, Henry A. Wallace founded Hi-Bred Corn (later known as Pioneer Hi-bred), the first company to commercially produce and sell hybrid maize seeds. It is still one of the leading companies today (as part of Corteva Agriscience). Currently, the majority of maize varieties in the U.S. and worldwide are hybrids. In the U.S., average maize yield has increased about 7-fold since the early 1900s, rising from approximately 1.4 Mg ha^−^¹ to 11.7 Mg ha^−^¹ by 2025, while planting density increased from about 25,000 to 90,000 plants ha^−^¹, a 3.5-fold increase (Duvick, 2005a; Nielsen, 2023; USDA, 2025).

Changes in agriculture significantly shaped the development of modern hybrids over the past 100 years, such as the use of fertilizers and pesticides, and mechanization. Key drivers of yield gains in modern hybrids include tolerance to higher planting densities (Assefa et al., 2018) and resistance to drought and lodging (Campos et al., 2006; Cooper et al., 2014), which led to changes in plant stature (Hammer et al., 2009; Borrás and Vitantonio-Mazzini, 2018; Wang et al., 2020; Ordóñez et al., 2024). Most recently, breeding programs have focused on increasing harvest index (Ruiz et al., 2023; Shao et al., 2024), leading to the development of short-stature maize varieties capable of tolerating even higher planting densities (Kosola et al., 2023). In contrast, the potential yield per plant has not changed (Tokatlidis and Koutroubas, 2004; Duvick, 2005b; Lee and Tollenaar, 2007). These advances have emerged during the period of rapidly changing climate conditions.

During the last century, atmospheric CO_2_ levels significantly increased from around 280 to 420 parts per million (ppm) (Tans and Keeling, 2016; Lan et al., 2023). The Mauna Loa Observatory in Hawaii recorded CO_2_ levels of 428 ppm in March 2025 (https://gml.noaa.gov). If current emission trends continue, CO_2_ concentrations are projected to exceed 540 ppm by the year 2100 (Meinshausen et al., 2011; Cui et al., 2024). Global surface temperatures have increased by more than 1 degree Celsius (°C) relative to the late 19^th^ century pre-industrial average, accompanied by more frequent extreme weather events (Coumou and Rahmstorf, 2012; Seneviratne et al., 2021; IPCC, 2022 and IPCC, 2023). These environmental changes pose a challenge to maize productivity (Gong et al., 2015; Ainsworth and Long, 2021; Rezaei et al., 2023). The increased frequency of droughts, floods, and storms can severely damage crops (Webber et al., 2018). Changes in rainfall distribution can result in water stress (Kim and Lee, 2023), while higher temperatures (Lesk et al., 2022) can accelerate plant development, shorten the growing season, leading to lower yields (Jägermeyr et al., 2021).

In maize, a C_4_ plant, mesophyll cells capture CO_2_ and incorporate it into four-carbon compounds, which are transported to bundle-sheath cells Vanaja et al. (2024). There, CO_2_ is released for assimilation by RuBisCO in the Calvin cycle, enhancing photosynthesis, especially under high temperatures and light conditions (Bouvier et al., 2024; Zafar and Bailey-Serres, 2025). Stomata, microscopic pores on lower leaf surfaces, are important for gas exchange and thus directly connected with multifactorial climate change (Hepworth et al., 2018; Gray and Dunn, 2024). In grasses, stomatal development is now understood to be environmentally plastic, responding to elevated CO_2_, drought, and temperature in ways that are relevant to crop adaptation under climate change (Chua and Lau, 2024; Chen et al., 2024). By opening, they enable CO_2_ uptake for photosynthesis and drive transpiration, which maintains water and nutrient flow from roots to shoots. Under limited soil moisture, evaporative demand can exceed the plant’s capacity to supply water. Leaf water potential declines, turgor is lost, and stomata begin to close, increasing stomatal resistance (r_s_) to CO_2_ diffusion (Dittberner et al., 2018; Buckley, 2019). This closure conserves water but restricts CO_2_ uptake, reducing photosynthesis, carbon gain, and growth (Sage, 2004; Lawson and Blatt, 2014). Both stomatal pore area and duration of stomatal opening are key determinants of plant water-use efficiency (WUE) (Henry et al., 2019; Barl et al., 2025). In maize, elevated CO_2_ levels (550 and 700 ppm) increased stomatal density while decreasing size, conductance, and transpiration, enhancing water-use efficiency (WUE) (Sonmez et al., 2023; Khan et al., 2024). However, direct yield gains under elevated CO_2_ are often limited in maize because C4 photosynthesis is already near saturation under current ambient CO_2_, whereas C3 plants generally show a stronger direct photosynthetic response. Elevated CO_2_ does not directly boost photosynthesis or yield under normal conditions; benefits are mainly indirect, aiding water conservation during drought by reducing stomatal conductance (Zhao et al., 2025), with potential yield increases expected under dry conditions (Leakey et al., 2009; van der Kooi et al., 2016).

The unique Pioneer ERA hybrids span 100 years of maize hybrid breeding and offer an opportunity to study changes in stomatal traits over this period. ERA studies (long-term breeding) have focused on overall yield improvement, and correlated traits, such as genetic gain, high planting density (Duvick, 2005a, 2005b), nitrogen (Haegele et al., 2013; York et al., 2015; DeBruin et al., 2017; Mueller et al., 2019), water and radiation use efficiency (Messina et al., 2022), and harvest index (Campos et al., 2006; Cooper et al., 2014; Kosola et al., 2023). Duvick and Cassman (1999) linked historical yield gains to reduction in tassel size and increases in stay-green traits. Campos et al. (2006) and Reyes et al. (2015) demonstrated that modern hybrids have shorter anthesis-silking intervals and experience less barrenness under drought stress (Smith et al., 2014). Over the past eight decades of breeding, Pioneer maize hybrids developed smaller root systems, which are likely to facilitate higher plant population densities (Rinehart et al., 2024), but a water capture capability similar to older varieties with larger root systems (Messina et al., 2022). Perez et al. (2019) found that European hybrids exhibited reduced early leaf area but little change later in the growth period. Overall, gains in leaf area index were primarily driven by increased planting density, while hybrid improvements enhanced density tolerance (Kalogeropoulos et al., 2024). The average leaf angle changed as the leaves became more erect (Elli et al., 2023). Maize water-use efficiency and harvest index have increased over the past decades (Rotundo et al., 2025). While century-long breeding hybrids have been intensely studied for agronomic traits, little is known regarding changes at the cellular level, because prior studies focused more on stress responses, development, and physiology than on release-year trends.

This study quantifies century-long changes in maize stomatal traits. We investigated stomatal traits in maize seedlings of 27 hybrids grown under controlled conditions. We evaluated stomatal length, width, size, total stomatal pore area (pore-to-leaf area), and leaf area for 27 Pioneer maize hybrids released between 1920 and 2022. Our objectives were to (a) quantify whether and how stomatal traits were changing over the past century, (b) assess the relationship between stomatal traits and changing environmental conditions in the past 100 years, and (c) discuss implications of stomatal trait changes for guiding future maize breeding strategies.

## 2 Materials and methods

### 2.1 Plant materials

We used a total of 27 genotypes representing the period of 1920 to 2022 including 14 historic Pioneer ERA genotypes (1920-2011) and 13 modern hybrids (2013-2022). Hybrid seeds (**Table 1**) were provided by (Pioneer brand) Corteva Agriscience.

**Table 1:** Maize hybrids used in this study, their year of release, and corresponding historic ERA and modern.

Hybrid identification code in the study	Hybrid name	Year of commercial release
Historic ERA (1920-2011)		
PHI01	Reid	1920
PHI02	307	1936
PHI03	352	1946
PHI04	354	1953
PHI05	354A	1958
PHI06	3376	1965
PHI07	3366	1972
PHI08	3382	1976
PHI09	3378	1983
PHI10	3379	1988
PHI11	3394	1991
PHI12	33P67	1999
PHI13	33T59	2007
PHI14	P1151	2011
Modern (still in use or marketed) (2013-2022)		
PHI15	P1197A	2013
PHI16	P1366	2017
PHI17	P0574	2014
PHI18	P1197B	2014
PHI19	P1185	2015
PHI20	P0953	2021
PHI21	P0995	2021
PHI22	P1082	2021
PHI23	P1222	2021
PHI24	P1244	2018
PHI25	P1413	2022
PHI26	P1548	2020
PHI27	P1587	2020

The collection included Reid Yellow Dent; an open-pollinated cultivar commonly used by farmers in the central US Midwest before the advent of commercial hybrids. Reid Yellow Dent was released in 1920 and is similar to earlier cultivars (Reyes et al., 2026). Additionally, P1197A (YHR) and P1197B (AM) were reclassified in 2013 and 2014, respectively, as distinct herbicide- or pesticide-tolerant versions.

### 2.2 Growth chamber experiments

The study was carried out at Iowa State University’s Department of Agronomy in Ames, Iowa, during the years 2024 and 2025. A growth chamber (CONVIRON Flex; 3.6 m^2^ growth area, 635 mm growth height) was equipped with light-emitting diodes (LEDs) to deliver a light intensity of 1000 µmol m^-2^ s^-1^ at 152 mm from the light source. Light output was monitored with a Quantum Light Meter (Apogee Instruments, Logan, Utah, USA). A photosynthetic photon flux density (PPFD) of 742.8 µmol m^-2^ s^-1^ within the photosynthetically active radiation (PAR) range of 400–700 nm was applied to the 27 maize hybrids (1920-2022). Photon flux densities were measured in the same growth chamber for 3 runs as follows: 0.83 µmol m^-2^ s^-1^ in the ultraviolet (UV) range, 124.3 µmol m^-2^ s^-1^ (400–500 nm), 294.7 µmol m^-2^ s^-1^(500–600 nm), 323.8 µmol m^-2^ s^-1^(600–700 nm), and 159.1 µmol m^-2^ s^-1^ (700–780 nm). Growth chamber CO_2_ concentration was supplied with ambient air and averaged ~425-431 µmol mol^−^¹ or parts per million (ppm) over the experimental period, consistent with seasonal variability in ambient CO_2_.

The daytime and night-time temperatures were maintained at 25 °C and 15 °C, respectively, with a light cycle of 14 hours of light and 10 hours of darkness and a relative humidity of 65% ± 5%. The chamber was regularly monitored to maintain proper temperature, humidity, and light intensity, ensuring plants remained healthy and free from stress or disease. All plants received the same amount of tap water, applied as needed to keep the soil consistently moist without waterlogging.

For each of the 27 hybrids, nine plants were evaluated per experiment (averaged the three plants to obtain one block-tray experimental unit to avoid pseudo replication at the plant level). The study was repeated in three independent growth chamber experiments, each using a randomized complete block design with three replications (27 genotypes × 3 plants as smallest experimental unit (“plot”) in each block × 3 replicates). To minimize the effects of micro-environments, the positions of 3-plant entries in trays were randomized within each run. Planting trays (15-cell trays, 127 mm deep) were filled with moistened Berger BM7 bark mix soil containing a starter fertilizer, bark, coarse perlite, dolomitic and calcitic limestone, premium coarse peat moss, and a non-ionic wetting agent. Maize seeds were planted at a depth of approximately 20 mm.

### 2.3 Plant measurements and stomatal imaging

Plant measurements were taken 14 days after planting across all hybrids, when the seedlings reached the four to five leaf stages (V4-V5). Because emergence occurred at different times-unevenly across plants within genotypes and replications, the growth stage was used as a reference for measurements (14 days). The second leaf of fully expanded (**Supplementary Figure 7**) (continuing from the base) was selected for both leaf area (LA) and stomatal traits (size, density, length, and width) assessments (**Supplementary Figure 8**). The same leaf position was used across all hybrids during sampling. LA was calculated as leaf length x leaf width x 0.75 (Elings, 2000), where width was recorded at the widest point of the blade, and length measured from the base of the leaf blade to the tip.

For stomatal measurements, a thin layer of clear nail polish was applied to the abaxial (lower) epidermis at the widest point of the blade and allowed to dry completely. A strip of Scotch^®^ Transparent Tape (3M, MN, USA) was then pressed onto the dried area and peeled away, lifting a cast of the epidermis containing stomatal imprints (Wu and Zhao, 2017). The tape was mounted on a glass slide for microscopic observation.

Microscopy was performed using an Olympus BX40 microscope equipped with a high-definition camera, trinocular viewing head, C-mount adapter, and 40 x/0.65 Ph_2_ ¥/0.17 objective lens and 10x ocular lens oil-immersion objective. The microscope allowed real-time observation of stomatal impressions and facilitated image and video capture onto a removable SD card. Stomatal impressions were imaged at 40 x magnification with a 10 x ocular lens using a Moticam S6 camera and Motic Analysis 3.1 software. High-resolution images were analyzed to quantify average stomatal size, pore area (µm^2^), length (µm), width (µm), and density (number of stomata per mm^−^²). Stomatal density per LA was estimated using the size of the imaged field at the specified magnification and the measured leaf area. All stomatal images from the 1920–2022 hybrid dataset were manually reviewed to eliminate false positives and false negatives. Pixel-based measurements were converted to micrometers (µm) using a predetermined scale factor derived from a calibrated micrometer slide. Final stomatal dimensions and densities were used for statistical analyses.

### 2.4 Total stomatal pore area calculation

The total stomatal pore area per leaf was estimated for each genotype as: total stomatal pore area (µm² leaf⁻¹) = leaf area (*mm*^2^) × stomatal density (stomata no. *mm*^-2^) × stomatal size (µm² stomate⁻¹).

In this study, stomatal density, average size and stomatal pore area were obtained from image analysis, and LA was measured on the second leaf (cm²), converted to mm² prior to calculation as described above. Because size was measured in µm², the calculated total pore area is expressed in µm². This calculation was used to evaluate changes in total pore area across hybrids released over the past century.

### 2.5 Data annotation and model training

A total of 192 maize isogenic line pairs (Yavuz et al., 2025) were used to train a model designed to predict stomatal position, orientation, and size on unrelated datasets. Images were derived from tape−mounted stomatal impressions captured by microscope, as described above. And that the 27 genotypes were completely unseen during training.

Stomatal features in each image were manually annotated using rotated-rectangle bounding boxes created with custom Python software to identify each stomate. The YOLOv8 Object Bounding Box (OBB) model (Jocher et al., 2023) was trained via transfer learning on an annotated stomatal dataset, employing data augmentation techniques such as rotations, translations, scaling, and mix-up to enhance robustness. Its performance was evaluated using metrics such as precision, recall, and mean average precision (mAP). Training was conducted over 100 epochs. The learning rate was dynamically adjusted during training to optimize model performance. The model’s performance on validation set achieved a precision of 0.86, a recall of 0.979, and mAP@50–95 of 0.535 (**Supplementary Figure 1**) (Jocher et al., 2023). Final box loss and classification loss were 0.78 and 0.45, respectively. Average Intersection over Union (IoU) for the validation set was 0.78. The average centroid distance was 14.1 pixels, and the average angle difference was 6.78 degrees. The data were separated using the ultralytics.data.split_dota module (Jocher et al., 2023) into 70% training, 15% validation, and 15% for testing.

### 2.6 Statistical data analyses

All statistical analyses were conducted using R software 4.4.3 (R Core Team, 2024) with RStudio (RStudio Team, 2024) (http://www.r-project.org). Traits were regressed using linear mixed-effects models, with hybrids and year of release as a continuous predictor (fixed effect), rep (block) as a random effect to evaluate changes over the period of the released-breeding period. Using a mixed model approach, the stomatal traits of each hybrid were expressed as: model and error structure; *i* = 1,…27, *H* index hybrids, *r* = 1,2,3 index growth-chamber runs (experiments), and *b* = 1,2,3 index blocks within runs.

For estimation of hybrid best linear unbiased estimates (BLUEs), hybrids were fitted as fixed effects, while growth-chamber run and block effects were fitted as random effects:


$${\text{y}}_{\text{ik}}=\mu +{\text{H}}_{\text{i}}+{\text{C}}_{\text{j}}+{\left(\text{H}\times \text{C}\right)}_{\text{ij}}+{\text{B}}_{\text{k}(\text{j})}+\epsilon_{\text{ijk}}$$

where y_ijk_ is the observed phenotypic value of the i-th hybrid in the k-th block nested within the j-th growth-chamber run; μ is the overall mean; H_i_ is the fixed effect of the i-th hybrid (continuous predictor hybrid effect); C_j_ is the random effect of the j-th growth-chamber run; (H × C) _ij_ is the random hybrid-by-run interaction; B_k_ (_j_) is the random effect of block nested within run; and ϵ_ijk_ is the residual error. The BLUE of each hybrid was extracted as the estimated marginal mean. The notation standards described by Li et al. (2021). For tests of hybrid main effect, we used the *HxC* interaction as the error term (denominator), which evaluates whether hybrid differences are consistent across replicated runs.

Long-term breeding trends were evaluated by regressing the adjusted genotypic value of each hybrid on its year of release:


$${\hat{\text{Y}}}_{\text{i}}=\beta_{0}+\beta_{1}{\text{X}}_{\text{i}}+\epsilon_{\text{i}}$$

where ${\hat{\text{Y}}}_{\text{i}}$ is the BLUE of the i-th hybrid for a given stomatal trait, X_i_ is the year of release of the i-th hybrid, β_0_ is the intercept, β_1_ is the regression coefficient describing the change in trait value per year of release, and ϵ_i_ is the residual error. Thus, each regression was based on 27 hybrid-level adjusted values, with one point representing one hybrid.

For variance-component estimation and repeatability, hybrids were fitted as random effects:


$${\text{y}}_{\text{ijk}}=\mu +{\text{H}}_{\text{i}}+{\text{R}}_{\text{j}}+{\text{B}}_{\text{k}(\text{j})}+{\left(\text{HR}\right)}_{\text{ij}}+\epsilon_{\text{ijk}}$$

where H_i_ is the random effect of the i-th hybrid, R_j_ is the random effect of growth-chamber run, B_k_ (_j_) is the random effect of block nested within run, (HR)_ij_ is the hybrid-by-run interaction, and ϵ_ijk_ is the residual error. Random effects were assumed to be normally distributed:


$${\text{H}}_{\text{i}}∼\text{N}(0,\sigma_{H}^{2}),\;{\text{R}}_{\text{j}}∼\text{N}(0,\sigma_{R}^{2}),\;{\left(\text{HR}\right)}_{\text{ij}}∼\text{N}(0,\sigma_{HR}^{2}),\;\epsilon_{\text{ijk}}∼\text{N}(0,\sigma_{e}^{2})$$

Because the experiment was conducted within a controlled-environment setting rather than across multiple field environments, reported entry-mean repeatability rather than broad-sense heritability. Entry-mean repeatability was estimated as:


$$R=\frac{\sigma_{H}^{2}}{\sigma_{H}^{2}+\frac{\sigma_{HR}^{2}}{r}+\frac{\sigma_{e}^{2}}{rb}}$$

where $\sigma_{H}^{2}$ is the hybrid variance, $\sigma_{HR}^{2}$ is the hybrid-by-run variance, σ²e is the residual variance, r is the number of growth-chamber runs, and b is the number of blocks within each run.

The calculation was performed using the lmer package in the *lme*4 R package (Bates et al., 2015) and *nlme* (Pinheiro et al., 2025). Figures were created for data visualization using the packages “ggplot2” (Wickham, 2016). Path analysis was performed to assess the total stomatal pore area using the `lavaan` package (Rosseel, 2012). The proposed model incorporated both direct and indirect relationships among the variables.

We analyzed the differences between historic hybrids (released from 1920 to 2011) and modern hybrids (released from 2013 to 2022). The significance of differences between means was assessed using two-sided Student’s t-tests at a 95% confidence level (α = 0.05). Pearson correlation coefficients were calculated to analyze relationships between the evaluated traits. Relationships between the year of release and traits were analyzed using linear least-squares regression. R^2^ calculations of marginal and conditional effects for the models were performed using the “performance” package (Lüdecke et al., 2021).

Climate proxy data, we analyzed year of release climate proxies (annual-mean atmospheric CO_2_ and annual temperature anomaly) from 1920 to 2022 (**Supplementary Figures 5**, **6**). (1958-present from Mauna Loa record, for earlier years from Law Dome ice-core CO_2_, harmonized). The atmospheric CO_2_ data were obtained from the Mauna Loa Observatory recordings accessible from the Earth System Research Laboratory (Climate Change: Atmospheric Carbon Dioxide at https://www.esrl.noaa.gov and accessed on April 15, 2025). Temperature anomalies, were sources from NOAA’s Merged Land-Ocean Surface Temperature dataset, using annually mean values for the same period (https://www.ncei.noaa.gov, accessed on April 15, 2025).

Annual atmospheric CO_2_ concentration and annual mean temperature anomaly corresponding to each hybrid release year were used as historical environmental covariates. Temperature anomaly was defined as the annual mean temperature deviation from the selected long-term baseline period. Both atmospheric CO_2_ concentration and average temperature anomaly were standardized before analysis. A composite environmental index was then calculated as the first principal component of standardized atmospheric CO_2_ concentration and standardized temperature anomaly:


$$\text{zEnv }=\text{ PC}1\;\left({\text{zCO}}_{2},\text{ zTemp}\right)$$

where zCO_2_ and zTemp are standardized annual atmospheric CO_2_ concentration and standardized annual temperature anomaly, respectively.

To evaluate whether leaf area mediated the relationship between the historical environmental index and stomatal traits, two linear mixed models were compared:


$$\text{M}1:\;{\text{Y}}_{\text{ij}}\;=\;{\text{β}}_{0}\;+\;{\text{β}}_{1}{\text{zEnv}}_{\text{i}}\;+\;{\text{u}}_{\text{j}}\;+\;{\text{ϵ}}_{\text{ij}}$$

$$\text{M}2:\;{\text{Y}}_{\text{ij}}\;=\;{\text{β}}_{0}\;+\;{\text{β}}_{1}{\text{zEnv}}_{\text{i}}\;+\;{\text{β}}_{2}{\text{zLA}}_{\text{i}}\;+\;{\text{u}}_{\text{j}}\;+\;{\text{ϵ}}_{\text{ij}}$$

where Y_ij_ is the stomatal trait value, zEnv_i_ is the standardized environmental index, zLA_i_ is standardized leaf area, u_j_ is the random effect of hybrid, and ϵ_ij_ is the residual error. The reduction in the zEnv regression coefficient from M1 to M2 was used to evaluate the extent to which leaf area explained the environmental association.

Path analysis was also performed to evaluate direct and indirect relationships among historical environmental variables, leaf area, and stomatal traits. The mediation model was specified as:


$$\text{zLA }=\text{ a}\left(\text{zEnv}\right)\;+\;{\text{ϵ}}_{1}$$

$$\text{Y }=\;c\prime \left(\text{zEnv}\right)\;+\text{ b}\left(\text{zLA}\right)\;+\;{\text{ϵ}}_{2}$$

Indirect effect = a × b

where a represents the effect of zEnv on leaf area, b represents the effect of leaf area on the stomatal trait, c′ represents the direct effect of zEnv on the stomatal trait after accounting for leaf area, and a × b represents the indirect effect.

## 3 Results

### 3.1 Stomatal trait distributions

Most stomatal traits followed near-normal distributions with minor skewness (**Supplementary Figure 2**). Stomatal size and density exhibited slight deviations, while total stomatal pore area showed a strong right-skewed distribution. Leaf area and stomatal length and width were normally distributed, displaying tight, symmetric variation. These descriptive patterns provide context for comparing historic ERA and modern hybrids. Composite traits displayed different characteristics. Stomatal size trait showed moderate skewness of about 0.8, with a kurtosis value a slightly peaked distribution. Stomatal density spanned from 35 to 54 mm^−^², clustered around 42 to 50 mm^−^², exhibited a skewness of -0.5. The kurtosis was 3.0. Leaf area ranged from 14 to 19 cm² and peaked around 18 to 21 cm². It exhibited a right skewness of 0.6 with kurtosis indicating a slightly platykurtic distribution. Total stomatal pore area ranged from 7.4 x 10^8^ to 1.26 x 10^9^ µm², showing a pronounced right skewness of 2.0 and high kurtosis, which points to a heavy right tail. Stomatal length ranged around 41 μm (from 38.9 to 45.5 μm) while the width was approximately 26.5 μm (from 25.3 to 28.3 μm). These traits displayed tight, symmetric distributions, with skewness values around 0 and kurtosis close to 3, indicating a normal distribution.

### 3.2 Comparison of historic ERA and modern hybrids for stomatal traits

Highly significant genetic variation was found for all stomatal traits across the 27 hybrids (Df = 26, *p* ≤ 0.001). Across all hybrids, stomatal size ranged from 1008.2 to 1269.7 μm², with a mean of 1098.4 μm². Stomatal density varied from 34.9 to 54.4 stomata/mm^−^², averaging 45.8 stomata/mm^−^². Stomatal length ranged from 38.9 to 45.5 μm, with a mean of 41.2 μm, while stomatal width was between 25.3 and 28.3 μm, averaging 26.5 μm. Total stomatal pore area ranged from 7.39 × 10^8^ to 12.60 × 10^8^ μm², with a mean of 9.57 × 10^8^ μm². Leaf area ranged from 14.0 to 29.0 cm², with a mean of 19.2 cm² (**Table 2**).

**Table 2:** Comparative statistics for stomatal traits in modern (≥ 2013) and historic ERA hybrids.

Trait	Genotype	Df	*p* value	Min	Max	Mean	Residual vs All (%)	D (Modern – Historic, % of All)
Stomatal size (μm²)	All	26	0.001	1008.2	1269.7	1098.4		
	Historic ERA	1	n.s.	1011.4	1269.7	1104.8	+0.6%	-1.2% (n.s.)
	Modern			1008.2	1193.2	1091.6	-0.6%	
Stomatal density (per mm^−^²)	All	26	0.001	34.9	54.4	45.8		
	Historic ERA	1	0.001	34.9	51.8	44.5	-2.8%	5.9% (*p* < 0.05)
	Modern			39.7	54.4	47.2	3.1%	
Stomatal length (μm)	All	26	0.001	38.9	45.5	41.2		
	Historic ERA	1	n.s.	38.9	45.5	41.4	+0.5%	-0.7% (n.s.)
	Modern			38.9	43.8	41.1	-0.2%	
Stomatal width (μm)	All	26	0.001	25.3	28.3	26.5		
	Historic ERA	1	n.s.	25.3	28.3	26.5	0.0%	-0.4% (n.s.)
	Modern			25.8	27.7	26.4	-0.4%	
Leaf area (cm^2^)	All	26	0.001	14.0	29.0	19.2		
	Historic ERA	1	0.001	14.9	29.0	20.5	+6.8%	-13.6% (*p* < 0.05)
	Modern			14.0	22.9	17.9	-6.8%	
Total stomatal pore area (μm²)	All	26	0.001	7.39 × 10^8^	12.60 × 10^8^	9.57 × 10^8^		
	Historic ERA	1	0.001	7.39 × 10^8^	12.60 × 10^8^	9.94 × 10^8^	+3.9%	-8.1% (*p* < 0.05)
	Modern			7.45 × 10^8^	11.84 × 10^8^	9.17 × 10^8^	-4.2%	

Df, Degree of freedom, statistically significant at *p* < 0.001, n.s. not significant.

Residual vs All (%): 100 × (group mean − All mean)/All mean. D (Modern − Historic, % of All): 100 × (Modern mean − Historic mean)/All mean. Positive D means Modern > Historic; negative means Modern< Historic.

**Figure 1:**
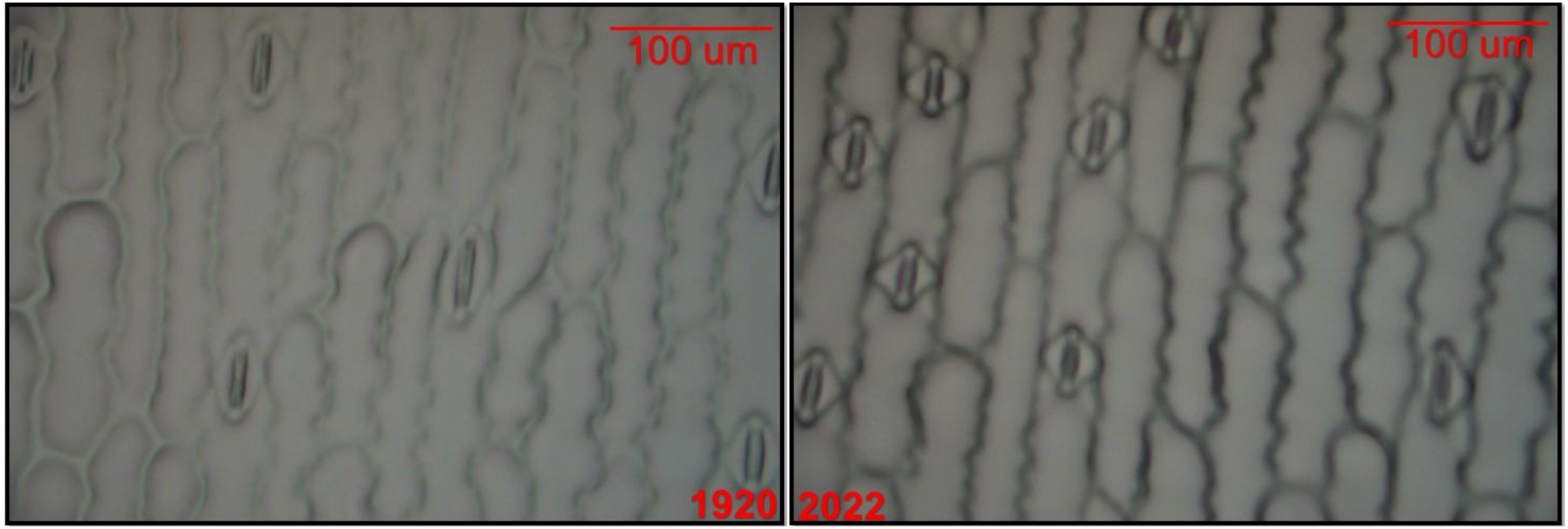
Microscopic images of stomata at 40X magnification from the hybrids released years 1920 and 2022, indicating that the same level magnification was used for both images. The size standards are measured in microns (µm). In 1920, the genotype exhibited a lower density but larger size, whereas in 2022, the genotype displayed a higher density but smaller size.

Representative microscopic images comparing stomatal impressions from the 1920 and 2022 hybrids are shown in **Figure 1**. The modern hybrids (2013 to 2022) had significantly (*p* < 0.05) increased stomatal densities (number per mm^−^²), and decreased leaf areas (cm²), and total stomatal pore areas (µm²), compared to historic ERA hybrids (1920 to 2011). Modern hybrids exhibited higher stomatal densities (mean: 47.2 stomata per mm^-^²; range: 39.7-54.4) than historic ERA hybrids (mean: 44.5 stomata per mm^-^²; range: 34.9-51.8), but smaller leaf areas (mean: 17.9 cm²; range: 14.0-22.9) compared to historic ERA hybrids (mean: 20.5 cm²; range: 14.9-29.0). In addition, the total stomatal pore area was smaller in modern hybrids (mean: 9.17 × 10^8^ μm²; range: 7.45 × 10^8^-11.84 × 10^8^ μm²) than in historic ERA hybrids (mean: 9.94 × 10^8^ μm²; range: 7.39 × 10^8^-12.60 × 10^8^ μm²). No significant differences were found in the size (μm²), length (μm), or width (μm) of individual stomatal between the two groups (**Table 2**).

**Table 2** compares modern and historic ERA hybrids on a percentage basis, as indicated in the row above. Stomatal size decreased by 1.2%, stomatal length by 0.7%, and stomatal width by 0.4%. While these changes are relatively small, stomatal density increased by 5.9%, and leaf area decreased by 13.6%. As a result, the total stomatal pore area is 8.1% lower.

### 3.3 Repeatability of stomatal traits

The entry-mean repeatability (R) of all stomatal traits ranged from 0.36 to 0.81 (**Table 3**). The highest repeatability (0.81) with narrow confidence interval (CI 0.53-0.85) was found for total stomatal pore area, while stomatal density showed the lowest repeatability of 0.36 (CI 0.12-0.61).

**Table 3:** Entry-mean repeatability (R) for stomatal traits.

Trait	Repeatability (R)	95% Confidence Interval (CI) Lower-Upper
Leaf area (cm²)	0.74	0.60-0.88
Stomatal size (µm²)	0.64	0.46-0.82
Stomatal density (per mm^−^²)	0.36	0.12-0.61
Stomatal length (µm)	0.68	0.51-0.85
Stomatal width (µm)	0.57	0.36-0.77
Total stomatal pore area (µm^−^²)	0.81	0.53-0.85

### 3.4 Trait correlations

**Figure 2:**
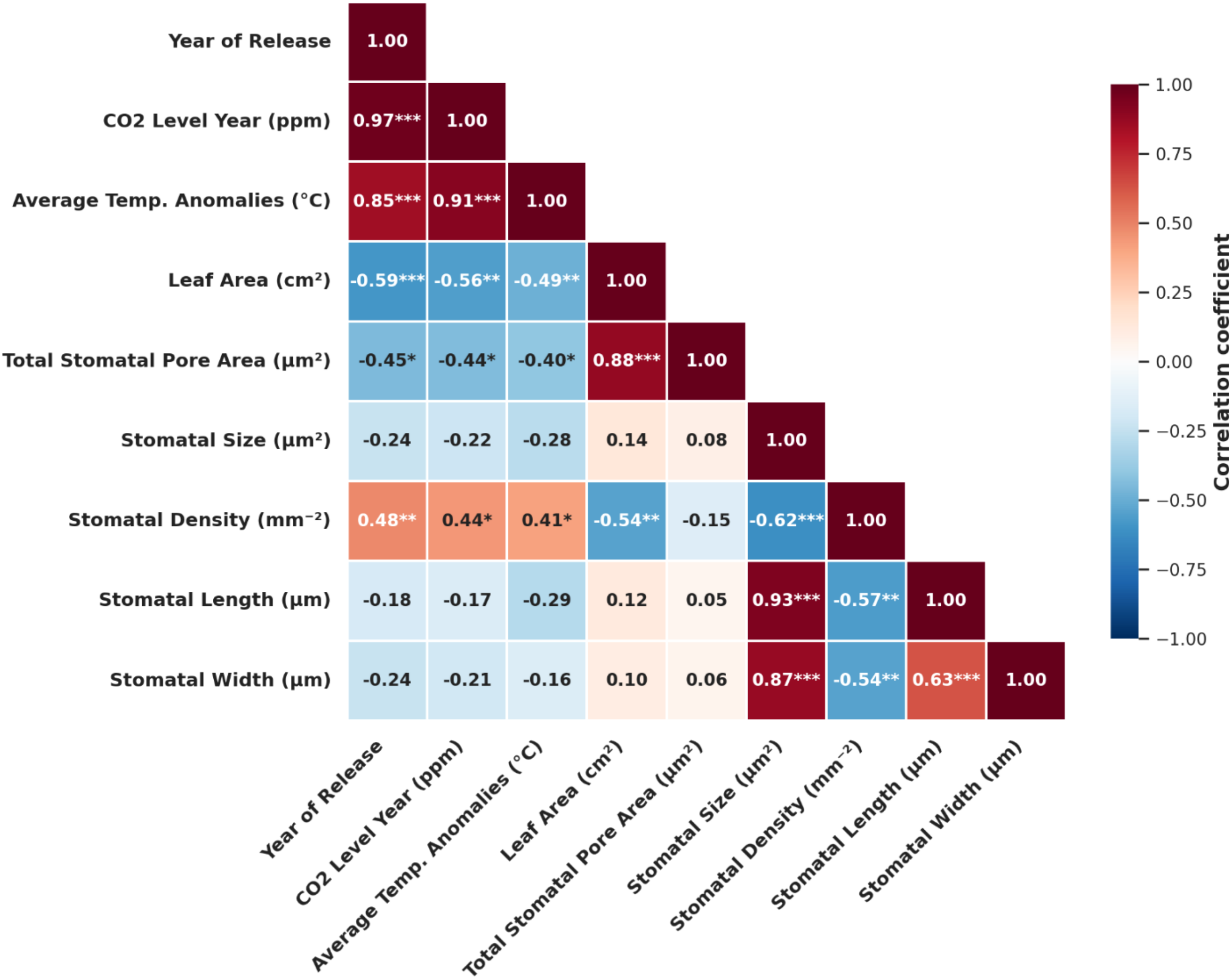
Pearson correlations were determined among all stomatal and climate traits. (* p< 0.05, ** p< 0.01).

Stomatal density was negatively correlated with stomatal traits such as stomatal size (r = -0.62, *p* < 0.05), length (r = -0.57, *p* < 0.05), width (r = -0.54, *p* < 0.05) and leaf area (r = -0.54, *p* < 0.05) (**Figure 2**). Stomatal size and length exhibited a strong positive correlation (r = 0.93, *p* < 0.05), as stomatal length increased, size also increased. Similarly, stomatal size and width showed a strong positive correlation (r = 0.87, *p* < 0.05), which was expected since size was dependent on both dimensions. Stomatal length and width had a positive correlation (r = 0.63, *p* < 0.05) stomatal tended to grow proportionally in both dimensions. Stomatal size showed a negative correlation of r= -0.22 with atmospheric CO_2_ concentration in parts per million (ppm) at the time the hybrid was released, whereas stomatal density exhibited a positive correlation of r= 0.44, *p* < 0.05. Additionally, stomatal size had a negative correlation of r= -0.28 with average temperature, while stomatal density had a positive correlation of r= 0.41 *p* < 0.05. Furthermore, the total stomatal pore area negatively correlated with both atmospheric CO_2_ concentration (r= -0.44, *p* < 0.05) and average temperature anomalies (°C) (r= -0.40, *p* < 0.05). Rising CO_2_ levels and increasing temperatures (°C) correlated with stomatal traits. These findings demonstrated a clear correlation with the atmospheric CO_2_ concentration and temperature changes in the hybrid’s year of release (**Figure 2**).

### 3.5 Trends for stomatal traits over time

**Figure 3:**
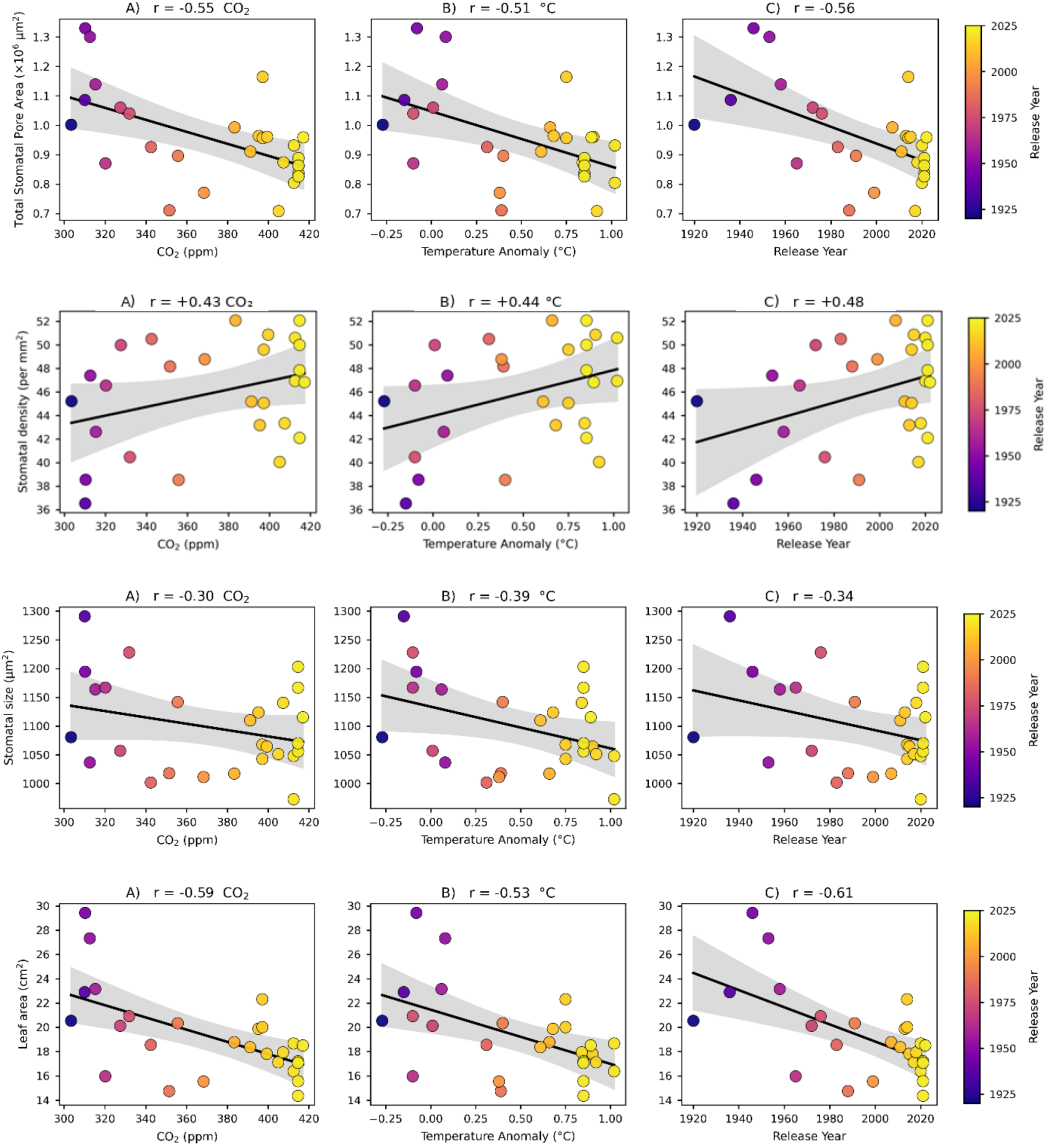
illustrates the relationships between total stomatal pore area, stomatal density, stomatal size, and leaf area relative to **(A)** atmospheric CO_2_ concentration, **(B)** mean temperature anomaly (°C), and **(C)** the year of release. This figure reflects long-term breeding trends in stomatal traits of 27 maize hybrids. Each point represents the adjusted genotypic value (BLUE) of a hybrid, estimated using a linear mixed model. The regression lines indicate the linear relationship between the year of hybrid release and the adjusted stomatal trait value, and they include 95% confidence intervals.

We observed that total stomatal pore area on the 2^nd^ leaf decreased with the year of release (**Table 2**). The trend from 1920 to 2022 (r= -0.56, r^2^ = 0.20; *p* < 0.05), y = -1992 x + 4.92 × 10^6^ also shows a significant decline (**Figure 3**). For each year, the total stomatal pore area decreased by approximately 1992 um^2^, which corresponds to an annual decline of about 0.02% relative to the initial mean value.

From 1920 to 2022 distinct trends were observed for stomatal traits of 27 hybrids. Stomatal size shows a non-significant negative correlation (r = -0.34), while stomatal density increased significantly over this period (r = 0.48, *p* < 0.05), indicating an increase in the number of stomatal per unit area.

Conversely, the total stomatal pore area exhibited a significant negative correlation with the year of release. Modern hybrids displayed a decrease in total stomatal pore area since 1920. Additionally, there is a strong negative correlation between stomatal size and density (r = -0.61, *p<* 0.05).

Modern hybrids have smaller stomatal size but a higher stomatal density compared to historic ERA hybrids. Thus, modern hybrids feature increased densities, but a smaller pore area than older hybrids. Moreover, plant leaf area decreased over the past 100 years.

We found a negative correlation between the total stomatal pore area of the entire leaf, CO_2_ concentration (r= -0.55, *p* < 0.05), and a negative association with temperature anomaly (°C) (r= -0.51). Additionally, stomatal density increased slightly with rising CO_2_ levels, with this relationship being statistically significant (*p* < 0.05). However, no significant relationship was observed between stomatal size and CO_2_ levels (**Figure 3**).

## 4 Discussion

The 27 hybrids used in our study are a small yet significant selection of maize germplasm, chosen for high agronomic importance in their respective eras between 1920 and 2022. In our study focusing on the 2^nd^ leaf, we assessed various stomatal traits. The entry-mean repeatability (R) of these traits ranged from 0.36 to 0.81 (see **Table 3**). While this range is suitable for analysis under controlled conditions, it is not known whether stomatal traits of the 2^nd^ leaf are predictive for other leaves on the same plant. We conducted a field study where we collected stomatal samples from the first leaf and the ear leaf of 10 different hybrids. While there was a significant difference between the first and ear leaf stomatal traits, they were closely correlated (r = 0.72). The measurements from the first leaf reliably predicted characteristics of the ear leaf (**Supplementary Table 7**). Long-term evolutionary changes in stomatal traits can be observed through field studies at varying CO_2_ concentrations. The study supports our findings, showing that stomatal density and number were low on new leaves but increased sharply during early development. Density stabilized once the leaves reached around 10 cm², while the stomatal number continued to increase with growth and also depend on drought conditions (Zhao et al., 2015). Importantly, stomatal density and size at different developmental stages (VT-R1) were not significant (Zhao et al., 2015) and our findings were closely correlated across genotypes (r = 0.72) (**Supplementary Table 7**). This suggests that stomatal traits measured under controlled conditions for a defined leaf in a panel of genotypes can be predictive for stomatal traits of other leaves of these genotypes. Controlled-environment conditions were used to reduce environmental variability, enabling consistent comparisons of stomatal traits among hybrids released over the decades.

We observed changes in maize stomatal traits in leading 27 hybrids representing subsequent decades over the past 100 years. Modern hybrids had a lower total stomatal pore area than historic ERA hybrids. Modern hybrids tended to have denser but smaller stomata with a reduced total stomatal pore area. The trend towards hybrids with a reduced total pore area suggests stomatal traits indirection selection to ongoing changes in global climate, including rising atmospheric CO_2_ levels, temperatures, and intensified drought conditions. Therefore, breeding-era associated trends under a common environment; climate variables are time-aligned context and strongly collinear with year.

A key question is whether increasing atmospheric CO_2_ concentration leads to a reduction in total stomatal pore area (increased stomatal density vs. decreased size) on maize leaves, as this would benefit the water balance of the plants. We observed changes in stomatal traits, including increased density (5.9%), decreased size (-1.2%) (not significant) and total stomatal pore area (-8.1%) per plant/leaf, compared to modern vs. historic ERA hybrids, resulting in an overall decline of about 0.02% per year over the past 100 years in the total stomatal pore area (**Table 2**). Despite modern hybrids having a smaller total stomatal pore area per plant, they are grown at a higher planting density, which enables similar yields while absorbing more CO_2_ per area due to the increased total number of plant leaves (Kalogeropoulos et al., 2024). Over the past century, the CO_2_ level has increased by 1.5-fold (from 280 to 426 ppm). This implies that significantly more carbon was converted into grain yield, facilitated by the rise in CO_2_ concentration. Interestingly, Kalogeropoulos et al. (2024) found a decrease in the average leaf area of individual plants: a decline of 0.33% per year from 1983 to 2017 and by 6.7% from 1936 to 2014 (Rinehart et al., 2024). Thus, although the average leaf area per plant has decreased (smaller leaves), the total leaf area per hectare has increased significantly. Today’s fields maintain or even exceed historic levels of total leaf area. Therefore, lower total stomatal pore area in modern hybrids may not indicate reduced functional capacity per se, but instead may reflect a shift toward more efficient stomatal regulation and water use (Rotundo et al., 2025), operating in conjunction with higher planting density and yield (Borras et al., 2026) and adjustments in whole-plant carbon allocation (Ruiz et al., 2026).

Our study demonstrated a close negative correlation between stomatal density and stomatal size (length and width), as well as leaf area in maize, aligning with short-term breeding studies (Zhao et al., 2015; Serna, 2022; Mano et al., 2023; Zhang et al., 2025). Considering stomatal density, size, and leaf area together offers more insight than analyses of any single trait over the past 100 years. Smaller stomata open and close more rapidly, improving control of gas exchange under water stress (Raven, 2014; Drake et al., 2013; Liu et al., 2019; Lawson and Vialet-Chabrand, 2019; Clark et al., 2022; Haworth et al., 2023). Gas-diffusion theory indicates that small, widely spaced pores are disproportionately more efficient because diffusion scales better with pore circumference than area (Bidwell, 1974).

We hypothesized that increased atmospheric CO_2_ and temperature °C resulted in smaller, denser stomata. Indeed, total stomatal pore area per leaf was negatively correlated with CO_2_ and temperature °C in year of release, linking size, density, and leaf area to climate and grain yield (Woodward, 1987; Driscoll et al., 2006; Ji et al., 2008; Franks and Beerling, 2009; Yan et al., 2017; Xu et al., 2016; Liang et al., 2023; Singh et al., 2025). In this study, structural equation modeling (SEM; **Supplementary Table 3**) indicated that elevated CO_2_ produced narrower and shorter stomata and reduced leaf area. The influences of CO_2_, temperature, and year on total stomatal pore area were mediated primarily through other traits, especially leaf area. CO_2_ was strongly positively correlated with temperature (r = 0.88). The year of hybrid release exerted a modest negative total effect on total stomatal pore area. Overall, CO_2_ had a negative total effect (r = -0.39), driven mainly by changes in stomatal density. These findings indicate that maize stomatal traits are associated with environmental changes, such as rising CO_2_ and temperature (**Supplementary Figure 3**; **Supplementary Table 3**-**5**). Because total stomatal pore area is a composite trait calculated from leaf area, stomatal density, and stomatal size, the path analysis should be interpreted as descriptive of covariance structure rather than as evidence of mechanistic causation. Meta-analyses indicated that stomatal frequency generally declines as CO_2_ rises but can increase with higher temperatures and drought, depending on species and conditions. At higher CO_2_, stomata tend to be smaller and more densely packed (Yan et al., 2017).

In maize, elevated CO_2_ (550 and 700 ppm) increased density but reduced size, conductance, and transpiration, improving WUE (Khan et al., 2024; Sonmez et al., 2023). These short-term studies support our long-term finding of increased stomatal density and decreased stomatal size in maize under rising atmospheric CO_2_ concentration conditions. However, these studies mentioned no yield gain benefit because C4 photosynthesis in maize is already near its CO_2_ efficiency maximum at 420 ppm, elevated CO_2_ does not directly stimulate photosynthesis or yield under non-stress conditions and projected benefits are mainly indirect (water-saving) under drought, with anticipated CO_2_-driven yield increases under dry conditions (Leakey et al., 2006, 2009; van der Kooi et al., 2016). Some studies nonetheless report yield gains or heat-stress mitigation and higher WUE at elevated CO_2_, and increased temperature can reduce stomatal density (Abebe et al., 2016; Lau et al., 2018; Khan et al., 2024; Bai et al., 2025).

Our findings are consistent with other long-term adaptation studies in C3 plants. While few long-term studies have been conducted, investigation of an herbarium in Arabidopsis resulted in a century long-term study showing declining stomatal density with rising CO_2_ (Lang et al., 2024). Similar long-term declines occurred in C3 angiosperm trees and ferns (e.g., *Pinus elliottii, Pinus taeda, Taxodium distichum*) over 150 years in Florida (Lammertsma et al., 2011). A 40% density decrease across eight temperate tree species over 200 years accompanied a CO_2_ rise from 280 to 340 ppm and was supported by controlled experiments (Woodward, 1987). In contrast, our results in maize showed increasing stomatal density, suggesting that long-term stomatal response trends (indirect selection) in C4 species may differ from those observed in many C3 species under climate change such as rising atmospheric CO_2_ and temperature (°C). Fossil and contemporary studies likewise associate density decline with rising CO_2_ (Hetherington and Woodward, 2003; Gienapp et al., 2008; Doheny-Adams et al., 2012; Vile et al., 2012; Zheng et al., 2013; Chen et al., 2024; Rovira et al., 2024; Klein et al., 2025). C3-C4 physiological differences may explain these contrasting patterns. C4 maize generally uses water more efficiently and regulates stomata rapidly under hot, dry conditions, whereas C3 plants thrive in cool, moist environments but are more sensitive to water loss (Ozeki et al., 2022; Way, 2012; Bertolino et al., 2019; Song et al., 2023). Elevated CO_2_ generally lowers stomatal density in C3 plants due to reduced need for CO_2_ diffusion to Rubisco (Osborne and Sack, 2012). In C4 plants, this response is weaker or absent because their carbon-concentrating mechanism buffers photosynthesis against CO_2_ changes (Maherali et al., 2002). Therefore, stomatal development in C4 plants is more influenced by hydraulic function, leaf cooling, and physiological coordination rather than CO_2_ supply alone (Taylor et al., 2018). This suggests different stomatal responses to elevated CO_2_ between C3 and C4 plants. While long-term C3 crop studies are very limited, short-term studies in wheat and soybean showed that density can fluctuate with drought and heat (Chua and Lau, 2024). A previous maize study involving 66 commercial hybrids released in Europe from 1950 to 2015 reported no changes in stomatal conductance or drought sensitivity, suggesting limited selective pressure on these traits over recent decades (Welcker et al., 2022). However, stomatal traits were not measured.

This ongoing indirect selection may not only have enhanced maize grain yield but may also plays a crucial role in addressing improved water use efficiency in modern production environments. Since 1950, rainfed maize yields in the USA corn belt have more than tripled without an increase in water inputs. The study revealed that the use of 61 ERA hybrids since 1934 has led to an increase in water use efficiency of 4.2% per year. This improvement is attributed to higher biomass productivity and better harvest index, with genetic advancements contributing 1.9% per year (Rotundo et al., 2025). Although water-use efficiency is a complex integrative phenotype, our results suggest that the smaller average stomatal pore area observed in modern hybrids may be one factor associated with improved water-use efficiency in maize, consistent with the study’s findings.

Over the past 100 years, significant changes have occurred in global climate conditions, plant density, water use efficiency, and maize yield. However, the per-plant grain yield remained relatively unchanged (Tokatlidis and Koutroubas, 2004; Duvick, 2005). We observed changes in stomatal traits, including an increase in stomatal density, a decrease in stomatal size, and consequently, a reduction in the total stomatal pore area per plant or leaf. These changes in stomatal traits appear to be a consequence of indirect selection for yield and are correlated with increasing atmospheric CO_2_ concentration levels and temperature. We propose that selection for yield stability, drought resilience, and tolerance to higher planting densities have inadvertently shaped stomatal traits over generations under climate change environments. Over the past century, the stomatal traits of maize evolved due to inadvertent selection linked to yield improvement over release years. These changes are consistent with long-term breeding progress and are statistically associated with release-year climate proxies.

Our findings showed that these changes generally favored increased stomatal density, reduced stomatal size, and smaller leaf area. These trends align with the biological expectation of a negative correlation between stomatal size and density, which likely results from geometric constraints and competition for epidermal space with other cell types, such as trichomes and glands (de Boer et al., 2016; Nunes, 2023; Chua and Lau, 2024). Based on our results, we propose that future maize breeding should prioritize smaller but more numerous stomata (density) per unit leaf area, which allows rapid adjustment of conductance and improved water-use efficiency. Efficient guard cell signaling, enabling quick responses to fluctuating conditions and light and vapor pressure deficit. These traits would not only improve resilience to climate change but also support higher yields. Intentional selection for stomatal traits offers a strategic advantage over inadvertent selection, particularly in drought-prone regions where water-use efficiency is critical.

### 4.1 Limitations of the study

This study provides a starting point for future maize breeding and physiology efforts aimed at improving stomatal efficiency and resilience under environmental-climate change, but several limitations should be considered. First, stomatal traits were measured only at the seedling stage and from a single leaf position. Although this improved developmental consistency, it may limit extrapolation across the canopy and into reproductive stages. However, the high correlation between first and ear-leaf stomatal traits observed in our field study (r = 0.72), together with evidence that stomatal density and size do not differ significantly between VT and R1 (Zhao et al., 2015), suggests that controlled measurements from a defined leaf can reasonably predict stomatal traits in other leaves and stages. Second, all plants were grown under a single contemporary chamber environment with modern ambient CO_2_, preventing us from disentangling long-term genetic differentiation from direct acclimation to historical atmospheric conditions. Still, our findings are broadly comparable to long-term studies in C3 species that reported declining stomatal density with rising CO_2_ (Lammertsma et al., 2011; Lang et al., 2024), was supported by controlled experiments (Woodward, 1987), although maize showed the opposite trend, indicating that adaptive responses may differ among species. Third, release year, atmospheric CO_2_, and temperature anomaly were strongly collinear over the last century, limiting clear separation of their independent effects (**Supplementary Figures 9**-**15**). These patterns are consistent with long-term genetic differentiation across release years, but the common-environment design may not allow formal separation of historical acclimation, adaptation, and correlated breeding change. Our findings align with observed declines in average leaf area (Kalogeropoulos et al., 2024; Rinehart et al., 2024) and support the hypothesis of long-term evolutionary selection rather than mere phenotypic plasticity, which only allows temporary adjustments in response to changing environments (Vinton et al., 2022). In addition, the relatively low repeatability (R) estimate for stomatal density suggests that environmental variation and/or limited genetic variance within this panel may contribute substantially to the observed phenotype. Future study integrating controlled historical CO_2_ treatments (direct acclimation to the historical CO_2_ and °C environments associated with their hybrids year of release), multiple developmental stages, and field validation across historical and modern germplasm should be needed to distinguish more directly genetic adaptation from plastic acclimation.

## Data Availability

The original contributions presented in the study are included in the article/**Supplementary Material**. Further inquiries can be directed to the corresponding author.
