# Supplementary material for: Trends in stomatal density and size in maize hybrids representing 100 years of long-term breeding for yield
Source: Front Plant Sci. 2026 May 13;17:1829321. doi: 10.3389/fpls.2026.1829321 (PMC13212258; doi:10.3389/fpls.2026.1829321)
Supplement: Supplementary file 1 [file Table1.docx]

**Additional Supplementary**


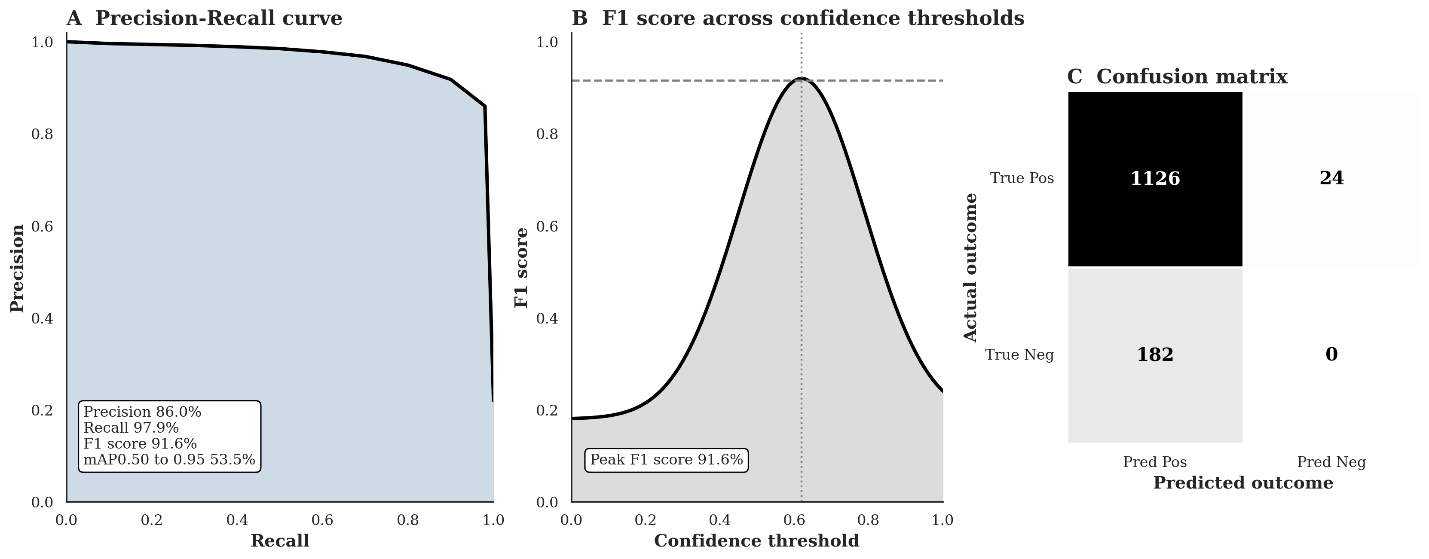


Supplementary Figure 1 shows a. F1 Score at Confidence Threshold Recall at Confidence Threshold, b. Precision at Confidence Threshold, and c. Recall at Confidence Threshold indicates the proportion of actual stomata correctly detected. A high recall rate indicates that there are few false negatives. F1 score is a harmonic mean of both precision and recall. It remains high only when both precision and recall are high. Average Precision at IoU Threshold of 0.50 metric. Average Precision at IoU Threshold of 0.50 metric shows the average precision when the Intersection over Union (IoU) threshold is set at 0.50. It implies that the model is considered correct if the predicted bounding box overlaps the true bounding box by at least 50%. mean average precision (mAP) evaluates model performance over a range of overlap thresholds, from loose (0.50) to strict (0.95) box matches in increments of 0.05. The moderate value reflects the expected decline in precision at stricter thresholds, where more exact bounding box alignment is required.

Validation of measurement accuracy for stomatal traits; the stomata detection model, YOLOv8 OBB, demonstrated strong performance. The recall, a metric that measures the model's ability to identify all positive instances in the dataset, was 97.9% (p ≤ 0.01). This indicates that the model correctly detected nearly all stomata present in the images, with very few cases missed. The precision was 86%, indicating that most of stomata identified by our model were correct although a small proportion were false positives. The F1 score was 91.6%. This indicates high reliability of our model for detecting stomata and avoiding misclassification. Mean Average Precision (mAP) is a reliable method for evaluating object detection models. It addresses challenges related to localization precision (IoU), confidence thresholds, and predictions across multiple classes. The mAP, calculated across IoU thresholds ranged from 0.50 to 0.95, is 53.5%. This indicates an expected decline in performance at stricter IoU thresholds, which is typical, as tighter matching presents greater challenges (Supplementary Figure 5). To assess the performance of the image-analysis pipeline, the purpose of this analysis was to quantify agreement and establish whether the pipeline could be used for higher-throughput trait extraction, rather than to validate the biological significance of the traits.

Supplementary Table 1 Summarizes the average of each stomatal trait of the second leaf after 14 days of growth.

| Table 1. The Average of Each Hybrid on Measured Traits of the Second Leaf After 14 Days. | | | | | | |
| --- | --- | --- | --- | --- | --- | --- |
|  |  | Stomatal Density | Stomatal size-area | Stomatal length | Stomatal  width | Total stomatal pore area |
| Release year | Genotype (hybrid) | Stomata mm^-^² | μm² | μm | μm | μm² |
| 1920 | Reid | 43.48 | 1056.85 | 39.26 | 26.70 | 9.36 × 10⁸ |
| 1936 | 307 | 34.89 | 1269.65 | 45.45 | 27.71 | 9.97 × 10⁸ |
| 1946 | 352 | 36.91 | 1171.23 | 43.68 | 26.63 | 12.48 × 10⁸ |
| 1953 | 354 | 45.76 | 1011.38 | 39.17 | 25.54 | 12.60 × 10⁸ |
| 1958 | 354A | 40.70 | 1139.09 | 41.20 | 27.52 | 10.57 × 10⁸ |
| 1965 | 3376 | 47.53 | 1177.94 | 42.01 | 27.90 | 8.95 × 10⁸ |
| 1972 | 3366 | 50.56 | 1067.74 | 41.43 | 25.48 | 10.96 × 10⁸ |
| 1976 | 3382 | 40.95 | 1238.78 | 43.73 | 28.27 | 10.68 × 10⁸ |
| 1983 | 3378 | 51.07 | 1012.08 | 39.73 | 25.38 | 9.67 × 10⁸ |
| 1988 | 3379 | 48.79 | 1028.70 | 40.58 | 25.29 | 7.39 × 10⁸ |
| 1991 | 3394 | 38.17 | 1144.96 | 42.67 | 26.73 | 9.06 × 10⁸ |
| 1999 | 33P67 | 48.28 | 1014.85 | 39.32 | 25.70 | 7.89 × 10⁸ |
| 2007 | 33T59 | 51.82 | 1020.52 | 38.90 | 26.12 | 10.15 × 10⁸ |
| 2011 | P1151 | 44.24 | 1113.21 | 42.03 | 25.98 | 9.41 × 10⁸ |
| 2013 | P1197A | 42.72 | 1127.16 | 40.81 | 27.53 | 9.79 × 10⁸ |
| 2014 | P0574 | 47.53 | 1043.57 | 39.97 | 25.98 | 9.87 × 10⁸ |
| 2014 | P1197B | 44.75 | 1069.87 | 40.79 | 26.09 | 11.84 × 10⁸ |
| 2015 | P1185 | 50.05 | 1066.51 | 41.02 | 25.86 | 9.90 × 10⁸ |
| 2017 | P1366 | 39.69 | 1052.10 | 40.35 | 25.85 | 7.45 × 10⁸ |
| 2018 | P1244 | 43.99 | 1135.09 | 42.19 | 26.73 | 8.62 × 10⁸ |
| 2020 | P1587 | 51.07 | 1090.56 | 41.12 | 26.40 | 8.76 × 10⁸ |
| 2020 | P1548 | 54.35 | 1008.20 | 38.86 | 25.83 | 10.03 × 10⁸ |
| 2021 | P0953 | 41.71 | 1169.97 | 42.09 | 27.68 | 8.03 × 10⁸ |
| 2021 | P0995 | 52.08 | 1193.15 | 43.81 | 27.13 | 8.23 × 10⁸ |
| 2021 | P1082 | 50.31 | 1064.93 | 40.57 | 26.10 | 8.68 × 10⁸ |
| 2021 | P1222 | 48.28 | 1054.84 | 40.63 | 25.80 | 8.73 × 10⁸ |
| 2022 | P1413 | 47.27 | 1114.55 | 41.97 | 26.40 | 9.34 × 10⁸ |

Supplementary Table 2 presents the regression coefficients (slope), coefficient of determination (R²), and statistical significance (p-values) for the relationship between measured stomatal traits and the year of hybrid release. Positive slopes indicate an increasing trend over time, while negative slopes suggest a decline. R² values reflect the proportion of variation explained by the year of release. Significant p-values (p < 0.05) indicate a statistically meaningful relationship between the trait and hybrid release year.

| Table 2. Parameters for the Linear Regression Analysis Between Measured Stomatal Traits and Year of Hybrid Release | | | | | | | |
| --- | --- | --- | --- | --- | --- | --- | --- |
|  |  | Correlation | Origin (1920 value) | Slope | R2 | p value | n |
| Total Stomatal pore area |  | -0.46 | 9.36 × 10⁸ | -1992 | 0.21 | ≤0.05 | 27 |
| Stomatal density |  | 0.46 | 43.5 | 0.077 | 0.21 | ≤0.05 | 27 |
| Stomatal size (area) |  | -0.25 | 1057 | -0.580 | 0.06 | ns | 27 |
| Stomatal length |  | -0.18 | 39.3 | -0.010 | 0.03 | ns | 27 |
| Stomatal width |  | -0.26 | 26.7 | -0.007 | 0.06 | ns | 27 |


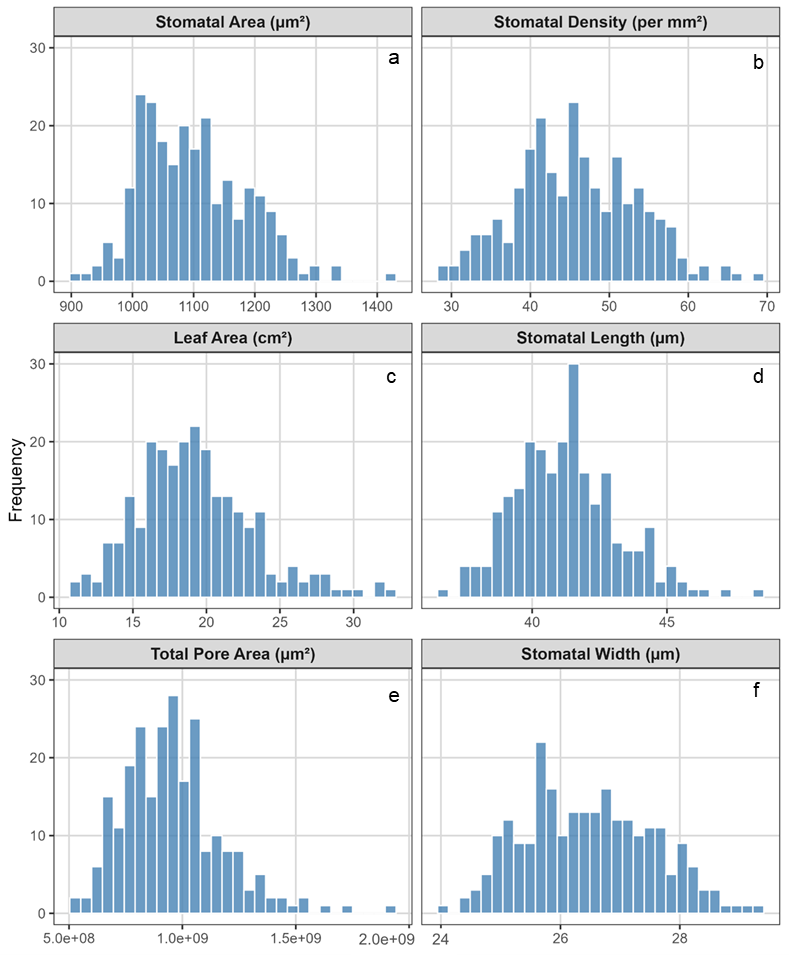


Supplementary Figure 2 Trait distributions across 27 hybrids: a. stomatal size (area); b. stomatal density; c. leaf area; d. stomatal length; e. total stomatal pore area; and f. stomatal width.


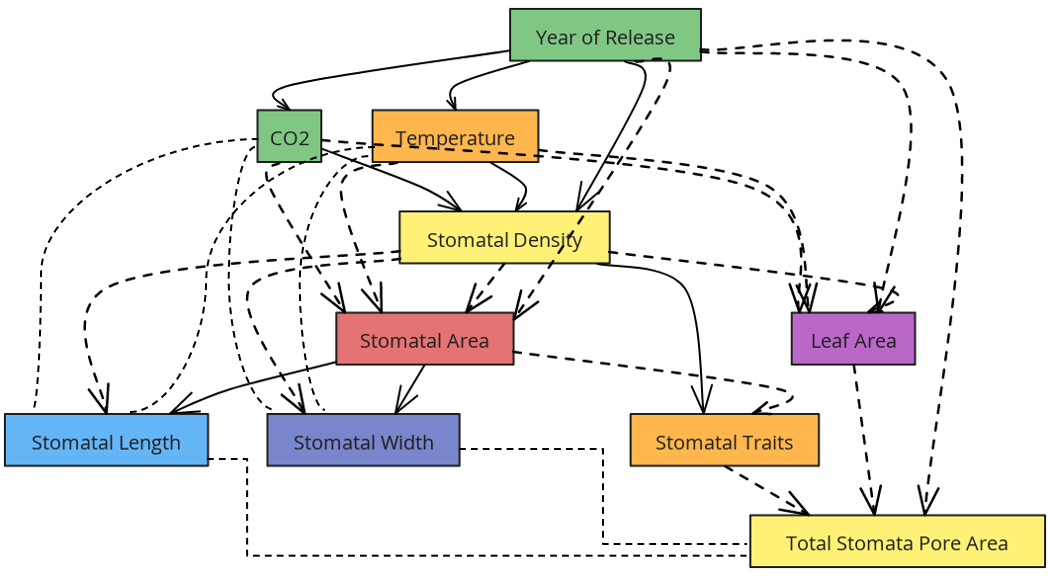


Supplementary Figure 3 Path analysis for total stomatal pore area. Positive correlations are represented by solid arrows, while negative correlations are indicated by dotted-line arrows. Stomatal traits showed the effect of density and area in the legend.

Path analysis

**Hypothesis 1:** Stomatal traits directly and positively affect total stomatal pore area.
**Hypothesis 2:** Leaf area indirectly affects total stomatal pore area through its influence on stomatal traits. **Hypothesis** 3: Year of release, increased atmospheric CO₂ concentration and temperature affect stomatal traits, leading to a reduction in total stomatal pore area.

Leaf area had the most significant impact on total stomatal pore area (d1 = -0.93, *p*≤ 0.001). Stomatal density contributed significantly (d2 = 0.39, *p*≤ 0.001). Stomatal length had a slight negative effect (d3 = -0.15, *p*≤ 0.05), while stomatal width had a modest positive effect (d4 = 0.17, *p*≤ 0.05). Increased CO₂ resulted in widened and shortened pores while leaf size was reduced, so its net effect was close to zero. Stomatal length decreased with rising temperatures, while stomatal width remained unaffected. Factors such as CO₂ levels, temperature, and year primarily influenced total stomatal pore area through these mediators, as their direct effects were minimal. Increased CO₂ and temperature exhibited a strong correlation (r = 0.88, *p*≤ 0.001). CO₂ was strongly negatively correlated with leaf area (r = -0.77), suggesting that higher CO₂ levels may be associated with smaller leaves.

Temperature anomaly showed only a weak, nonsignificant positive link to leaf area (r = 0.24). Larger leaves had lower stomatal densities but significantly greater total stomatal pore area (r = -0.57 and r = 0.81, respectively). Temperature's strongest indirect effect was via leaf area (+0.22), resulting in a total effect of (+0.13) on total stomatal pore area. The year of hybrid release had a net negative effect of (-0.18) because its pathway through leaf area (-0.73) outweighed the positive impact of stomatal density. The overall effect of CO₂ was negative (-0.39), mainly driven by the negative impact of stomatal density. Increased CO₂ impacted leaf area, giving an indirect negative influence on total stomatal pore area.

H₁: Stomatal Traits Adaptation: Year → Stomatal traits

Predictions: The effects of year and environmental conditions persist even after controlling for leaf area. The partial correlations are approximately equal to the total correlations, and direct effects are detectable.

H₂: Leaf-Mediated By-Product via leaf area): Year → Leaf area → Stomatal traits (mediation).

Predictions: The relationship from year to leaf area leads to stomatal traits through mediation. Adjusting for leaf area reduces the associations between year and stomatal traits, indicating that the path from year to leaf area is strong.


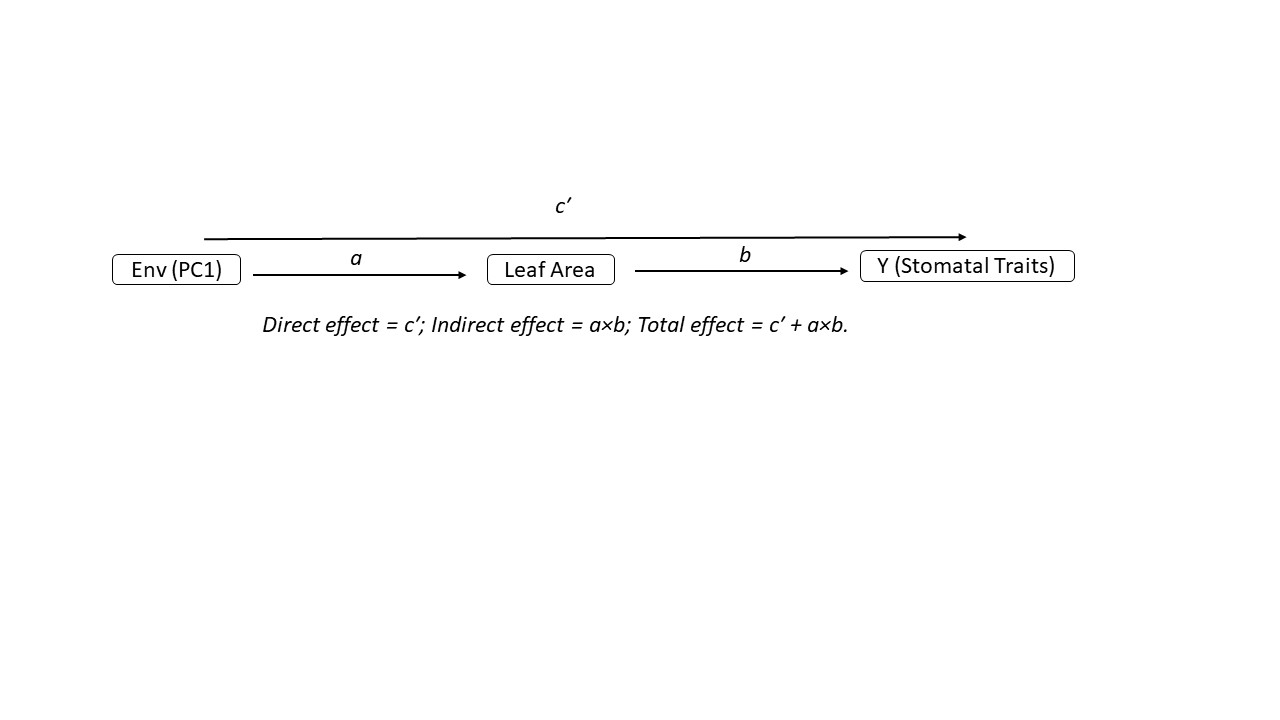


Supplementary Figure 4 Structural equation model (SEM) depicting direct (c′) and indirect (a×b) pathways from the environmental axis (PC1) to stomatal traits via Leaf area.

The environmental latent (Env) affects Leaf area (a-path), which in turn affects each outcome (b-path). Indirect effect = a×b; Direct effect = c′; Total effect = c′ + a×b. All coefficients are unstandardized; p-values as reported.

Supplementary Table 3 SEM summary including indirect and total effects.

| Trait | a (Env → LA) | b (LA → Trait) | Indirect | Direct (c′) | Total Effect (c′ + a×b) | Verdict |
| --- | --- | --- | --- | --- | --- | --- |
| Stomatal Density | -1.932  (p < .001) | -0.411  (p = .002) | 0.794 | 1.279  (p = .01) | 2.073 | Both direct and mediated |
| Stomatal Size | -1.932  (p < .001) | -0.187  (n.s.) | 0.362 | -17.23  (p = .006) | -16.87 | Direct only |
| Total Stomatal Pore Area | -1.932  (p < .001) | 39.8M  (p < .001) | -76.9M | 14.7M | -62.2M | Mediated |

Maize stomatal traits changed due to environmental (CO₂ and °C) response but total stomatal pore area, effects indirectly by decreased leaf area in maize hybrids representing 100 years of long-term breeding for yield. Leaf area was direct and indirect response to environment changed.


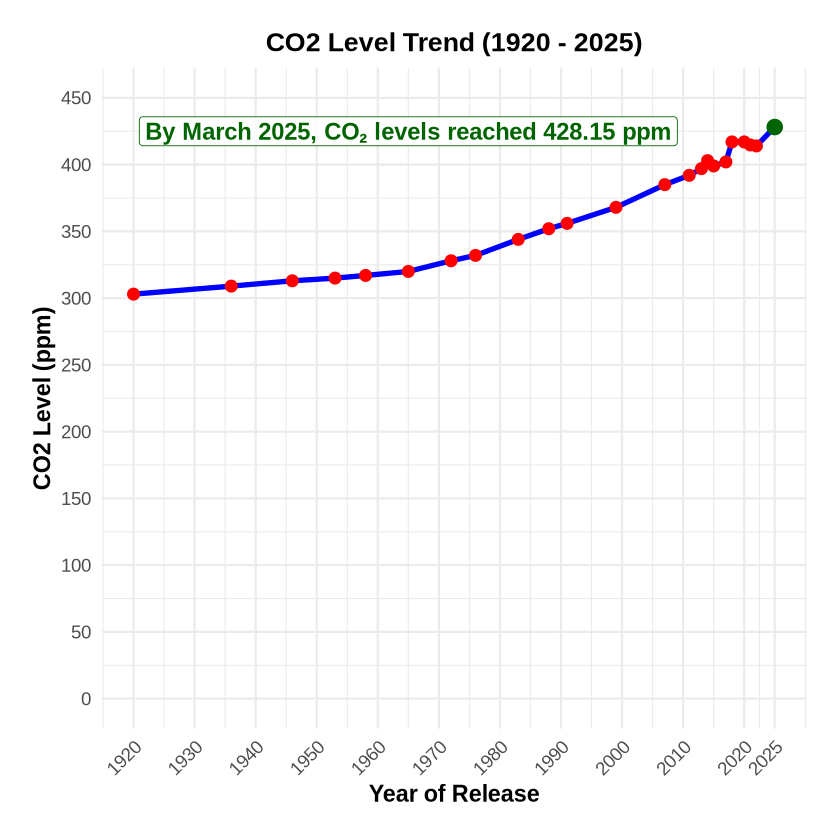


Supplementary Figure 5 Illustrates the last month of the year average carbon dioxide measured at Mauna Loa Observatory; Hawaii adapted from NOAA. The carbon dioxide data constitute the longest record of direct measurements of CO₂ in the atmosphere on Mauna Loa. NOAA. 2025. Climate change: atmospheric carbon dioxide. Earth System Research Laboratory. Available at: www.esrl.noaa.gov. Accessed: 4/15/2025.


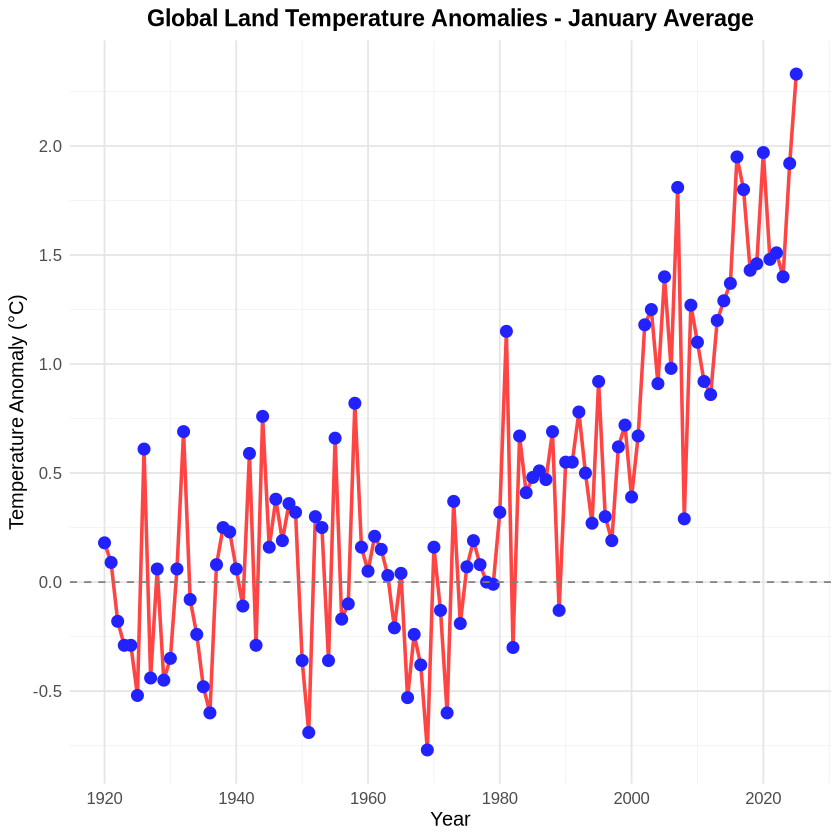


Supplementary Figure 6 Illustrates the average temperature anomaly (°C) for each January of the year, as measured by the NOAA Merged Land-Ocean Global Surface Temperature Analysis (Available at https://www.ncei.noaa.gov, accessed :4/15/2025).


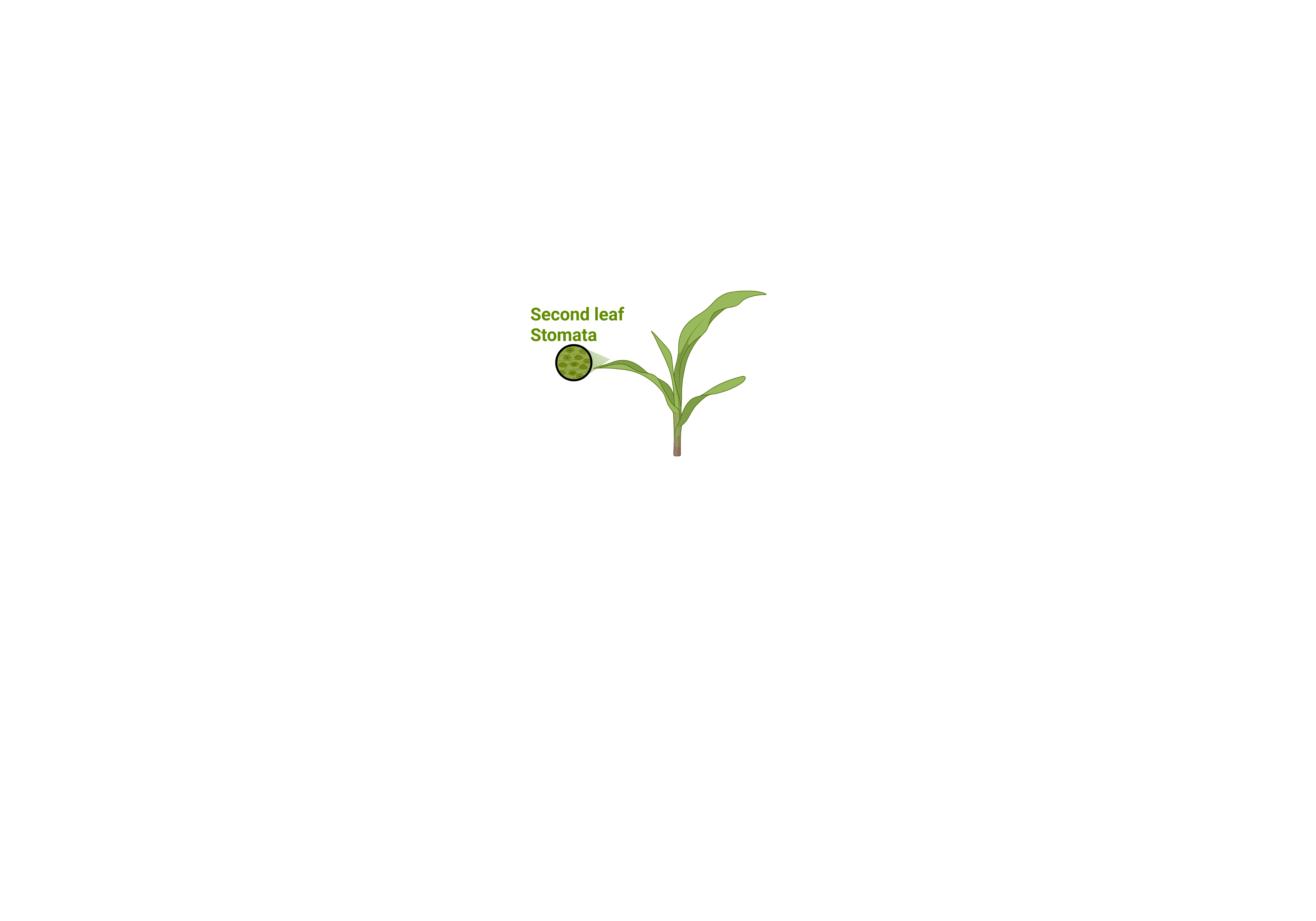


Supplementary Figure 7 the diagram illustrates a maize plant, specifically highlighting the second leaf where stomatal traits are measured. This is the second basal leaf that emerges shortly after the first leaf during the plant's development. An inset within the diagram shows a microscopic view of the stomata, emphasizing that measurements such as density, length, width, and area were taken from this location.


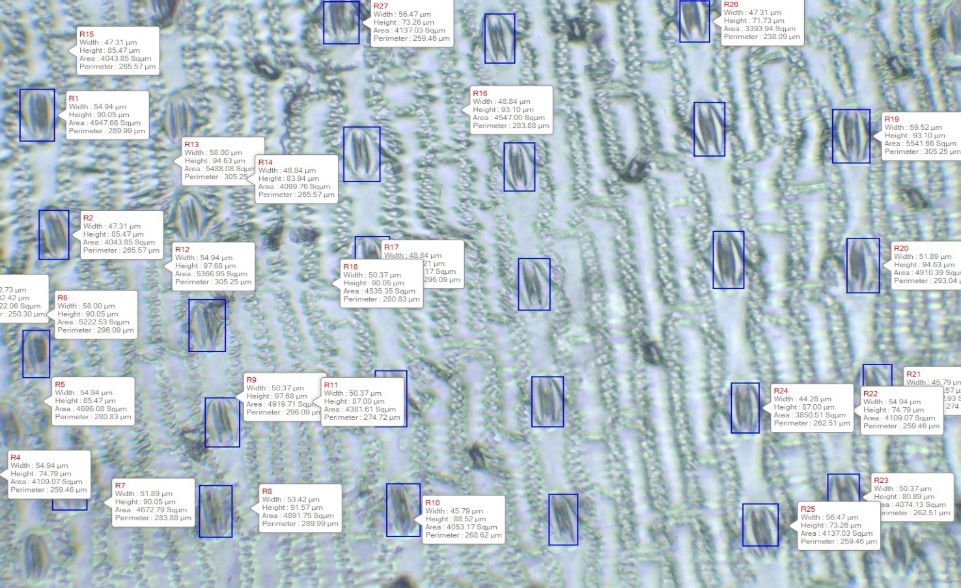


Supplementary Figure 8 presents a microscopic view of stomatal traits, including stomatal length (μm), which measures the distance from one end of the pore to the other, and stomatal width (μm), which is the measurement perpendicular to the length (short axis). Stomatal area-size (μm²) is defined as the overall area of the stomatal pore. Stomatal density (stomata/mm⁻²) is represented by displaying a defined surface area along with a count of the stomata within that area.

Supplementary Table 4 model fit (marginal and conditional R²) for M1 and M2, with differences (M2-M1).

| Outcome | R2 marginal M1 | R2 conditional M1 | R2 marginal M2 | R2 conditional M2 | Δ Marginal (M2-M1) | Δ Conditional (M2-M1) |
| --- | --- | --- | --- | --- | --- | --- |
| Stomatal Density | 0.08 | 0.38 | 0.08 | 0.37 | 0.00 | -0.01 |
| Stomatal Size | 0.04 | 0.67 | 0.04 | 0.67 | 0.00 | 0.00 |
| Stomatal Pore Index | 0.04 | 0.23 | 0.06 | 0.21 | 0.02 | -0.02 |
| Stomatal Length | 0.02 | 0.69 | 0.03 | 0.70 | 0.01 | 0.01 |
| Stomatal Width | 0.04 | 0.58 | 0.04 | 0.58 | 0.00 | 0.00 |
| Total Stomatal Pore Area | 0.08 | 0.36 | 0.51 | 0.57 | 0.43 | 0.21 |

Supplementary Table 5 Computed SEM effects (unstandardized).

| Outcome | a | b | Indirect (a×b) | Direct (c′) | Total Effect (c′ + a×b) |
| --- | --- | --- | --- | --- | --- |
| Stomatal Density | -1.932 | -0.411 | 0.794 | 1.279 | 2.073 |
| Stomatal Size | -1.932 | -0.187 | 0.362 | -17.228 | -16.866 |
| Stomatal Pore Index | -1.932 | -475.643 | 919.068 | 709.880 | 1628.948 |
| Total Stomatal Pore Area | -1.932 | 39.8M | -76.9M | 14.7M | -62.2M |

Abbreviations: SD, stomatal density; SS, stomatal size; SPI, stomatal pore index (SD×SS); TSPA, total stomatal pore area, SD×SS scaled to mm².

Supplementary Table 6 attenuation of zEnv, LMG fixed-effects contributions, and mediation verdicts.

| Trait | % Attenuation after LA | LMG (Env vs LA),  % of fixed R²) | SEM evidence for mediation | Final interpretation |
| --- | --- | --- | --- | --- |
| Stomatal Density | 11.6% (small) | Env 44% vs LA 56% | a: (p < .001); b: (p=.002)  indirect > 0 (0.80) | Both direct and mediated |
| Stomatal Size | −7.8% (no attenuation) | Env 90% vs LA 10% | a: (p < .001); b: n.s.  no indirect | Direct Env effect |
| Stomatal Length | 24.8% (moderate) | Env 70% vs LA 30% | a: (p < .001); b: n.s.  no indirect | Mostly direct |
| Stomatal Width | 7.9% (tiny) | Env 91% vs LA 9% | a: (p < .001); b: n.s.  no indirect | Direct env effect |
| Stomatal Pore Index | 33.8% (large) | Env 28% vs LA 72% | a: (p < .001); b: (p=.001)  large indirect (919) | Strong mediation via LA (+ direct) |
| Total Stomatal Pore Area | 100% (full) | Env 9% vs LA 91% | a: n.s.; b: (p < .001)  larger indirect | Full mediation via LA |

Maize stomatal traits changed due to environmental (CO₂ and °C) adaptation but total stomatal pore area, effects indirectly by decreased leaf area in maize hybrids representing 100 years of long-term breeding for yield. Leaf area was direct and indirect adaptation to environment changed. We observed significant declines in leaf area across release years (r²=0.30, *p*< 0.001), alongside increased stomatal density (r²=0.09, *p*< 0.001) and modest decreases in stomatal size (r²=0.013, *p*< 0.05). Leaf area was strongly and negatively associated with historical CO₂ and temperature (r²=0.29 and 0.20, respectively; *p*< 0.001). When accounting for CO₂ and temperature, the year of release had a significant effect on leaf area, suggesting that both long-term trends of CO₂ and temperature and the year of release hybrid contribute to changes in leaf area. This finding indicates that the mediation is incomplete, supporting a dual-mechanism model where both environmental changes and breeding-driven reductions in leaf area contribute to stomatal evolution. In mediation analyses, the year effect on density persisted after adjusting for leaf area indicating incomplete mediation and supporting a dual-mechanism model. The environmental axis (PC1) captured a balanced gradient of CO₂ and temperature, with loadings of 0.707 for each. Model fit comparisons revealed the largest marginal R² improvement for total stomatal pore area (M2), which increased by +0.43, aligning with the idea that leaf area mediates the composite stomatal pore area.

Supplementary Table 7 Relationship between an early first leaf and the ear leaf across 10 hybrids. Each point represents average one hybrid. Although leaf positions differed but the traits were positively correlated (r = 0.72; p < 0.01).

| Hybrid Name | First leaf | Ear leaf | Difference (Ear - First) | | |
| --- | --- | --- | --- | --- | --- |
| Hybrid 1 | 51.1 | 54.5 | 3.4 | | |
| Hybrid 2 | 55.0 | 82.2 | 27.2 | | |
| Hybrid 3 | 59.7 | 81.1 | 21.4 | | |
| Hybrid 4 | 55.2 | 54.8 | -0.4 | | |
| Hybrid 5 | 40.4 | 67.4 | 27.0 | | |
| Hybrid 6 | 59.6 | 63.9 | 4.3 | | |
| Hybrid 7 | 46.5 | 52.7 | 6.2 | | |
| Hybrid 8 | 55.3 | 87.8 | 32.5 | | |
| Hybrid 9 | 60.1 | 85.4 | 25.3 | | |
| Hybrid 10 | 47.0 | 48.9 | 1.9 | | |
| Trait | First leaf mean ± SE | Ear leaf mean ± SE | Difference (Ear - First) | r | n |
| Stomatal trait | 52.99 ± 3.2 | 67.87 ± 5.7 | 14.88 | 0.72 | 10 |


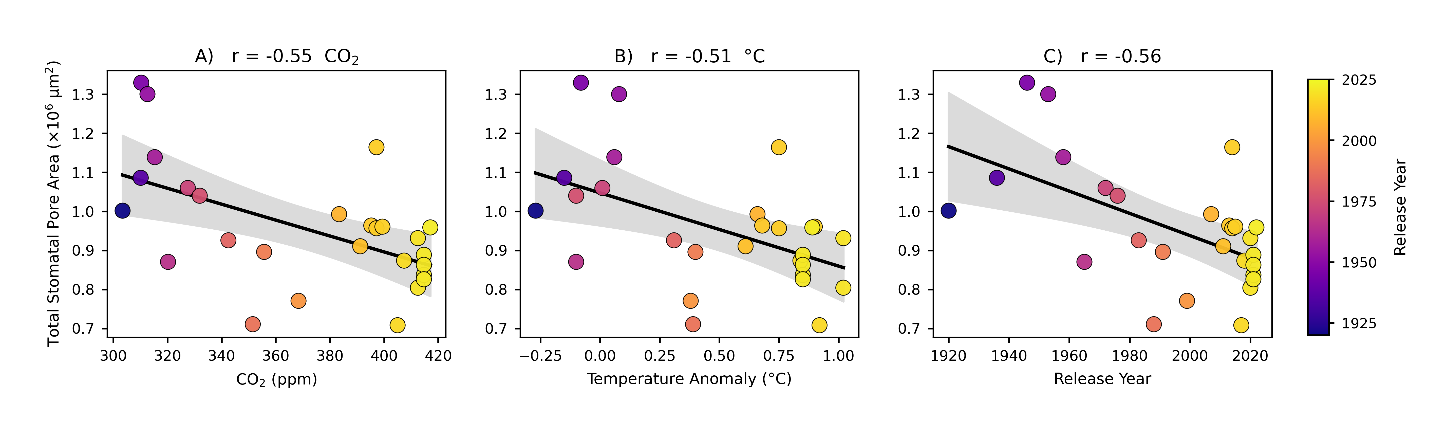
Supplementary Figure 9. Total stomatal pore area versus (A) atmospheric CO₂ (ppm), (B) mean temperature anomaly (°C), and (C) release year. Points are BLUEs observations colored by release year (A-B) or decade (C). Lines are linear regressions with 95% confidence bands.
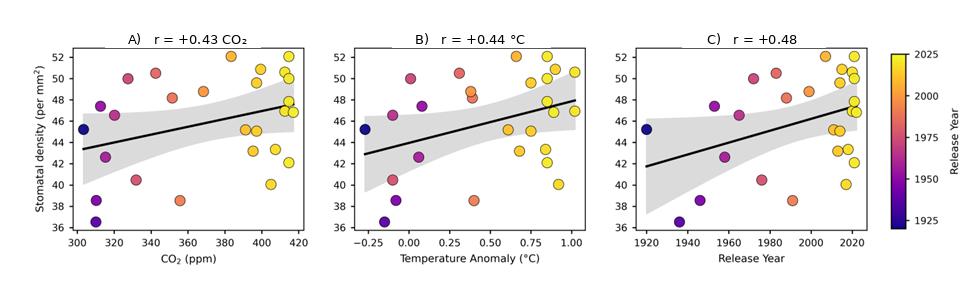
Supplementary Figure 10. Stomatal density versus (A) atmospheric CO₂ (ppm), (B) mean temperature anomaly (°C), and (C) release year. Points are BLUEs observations colored by release year (A-B) or decade (C). Lines are linear regressions with 95% confidence bands.
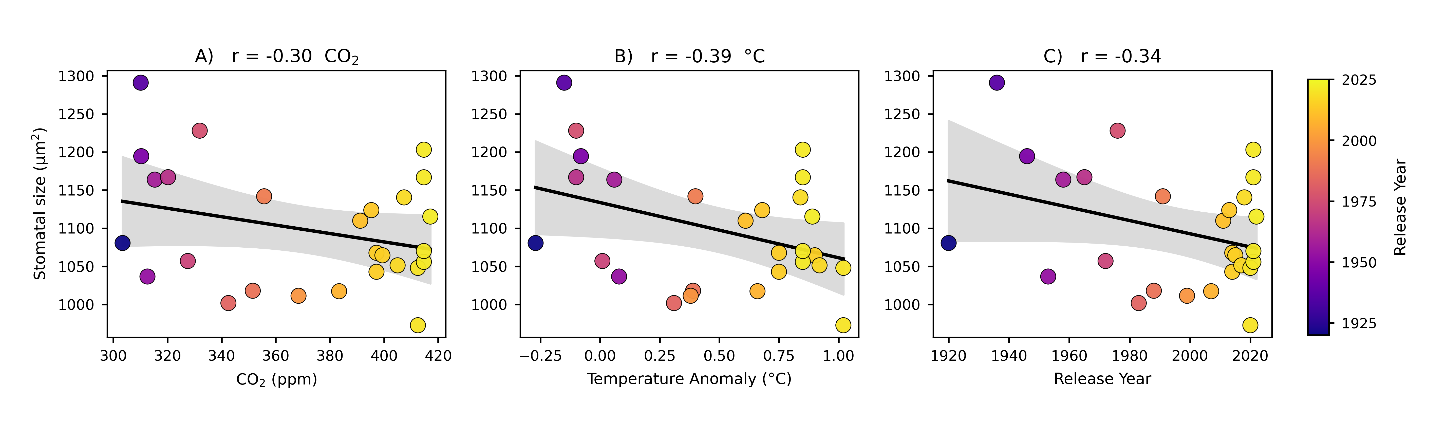


Supplementary Figure 11. Stomatal size versus (A) atmospheric CO₂ (ppm), (B) mean temperature anomaly (°C), and (C) release year. Points are BLUEs observations colored by release year (A-B) or decade (C). Lines are linear regressions with 95% confidence bands.


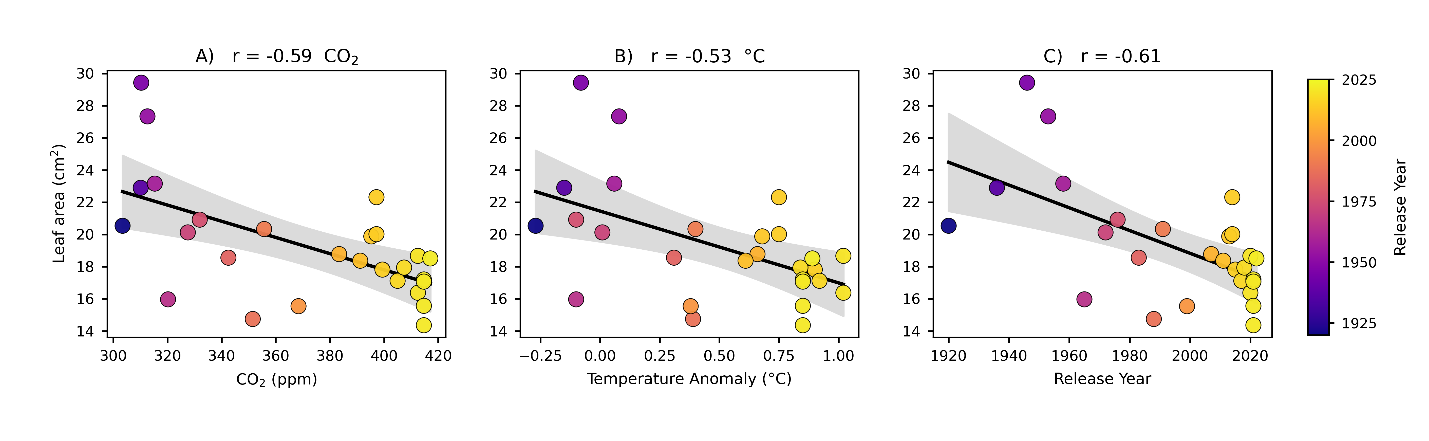
Supplementary Figure 12. Leaf area versus (A) atmospheric CO₂ (ppm), (B) mean temperature anomaly (°C), and (C) release year. Points are BLUEs observations colored by release year (A-B) or decade (C). Lines are linear regressions with 95% confidence bands.
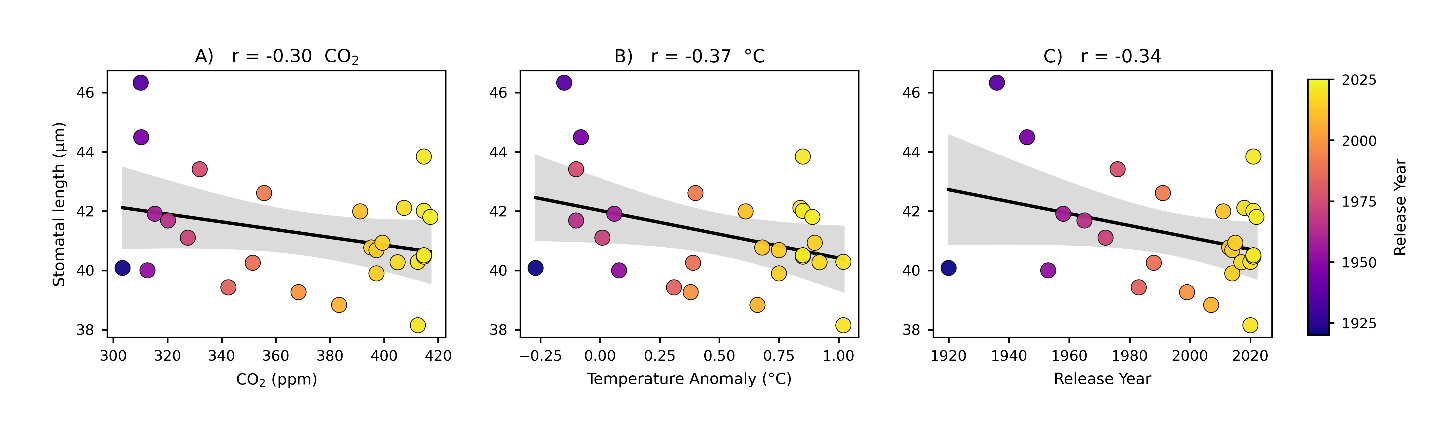
Supplementary Figure 13. Stomatal length versus (A) atmospheric CO₂ (ppm), (B) mean temperature anomaly (°C), and (C) release year. Points are BLUEs observations colored by release year (A-B) or decade (C). Lines are linear regressions with 95% confidence bands.
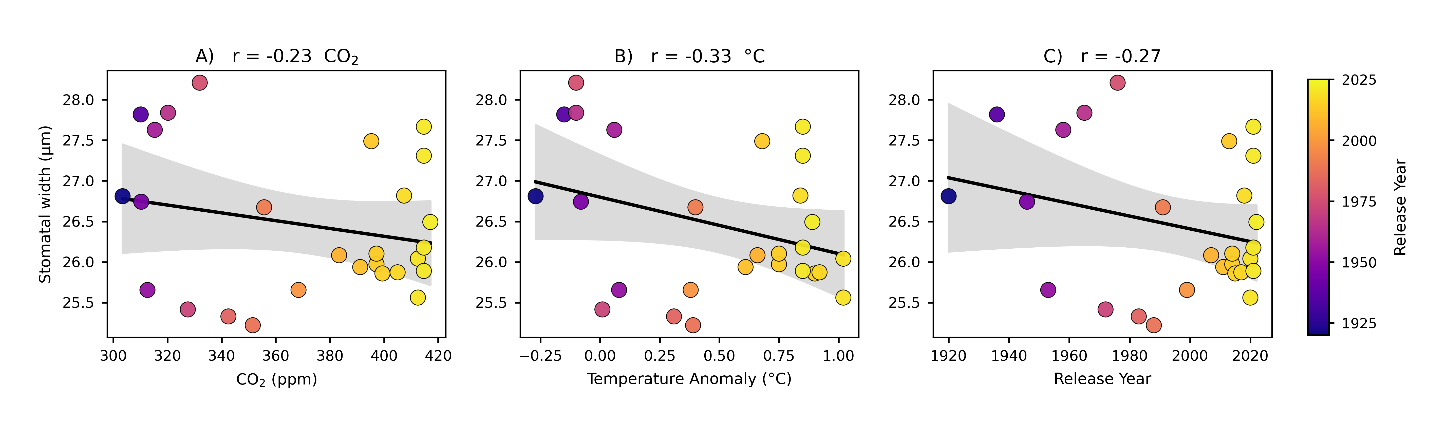


Supplementary Figure 14. Stomatal width versus (A) atmospheric CO₂ (ppm), (B) mean temperature anomaly (°C), and (C) release year. Points are BLUEs observations colored by release year (A-B) or decade (C). Lines are linear regressions with 95% confidence bands.
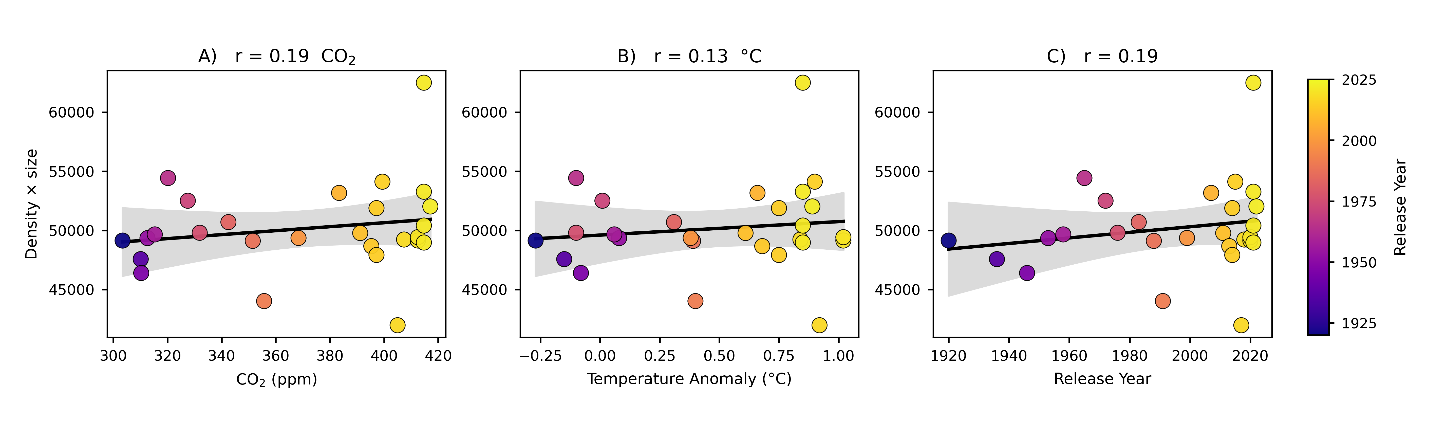


Supplementary Figure 15. Stomatal density x size versus (A) atmospheric CO₂ (ppm), (B) mean temperature anomaly (°C), and (C) release year. Points are BLUEs observations colored by release year (A-B) or decade (C). Lines are linear regressions with 95% confidence bands.
